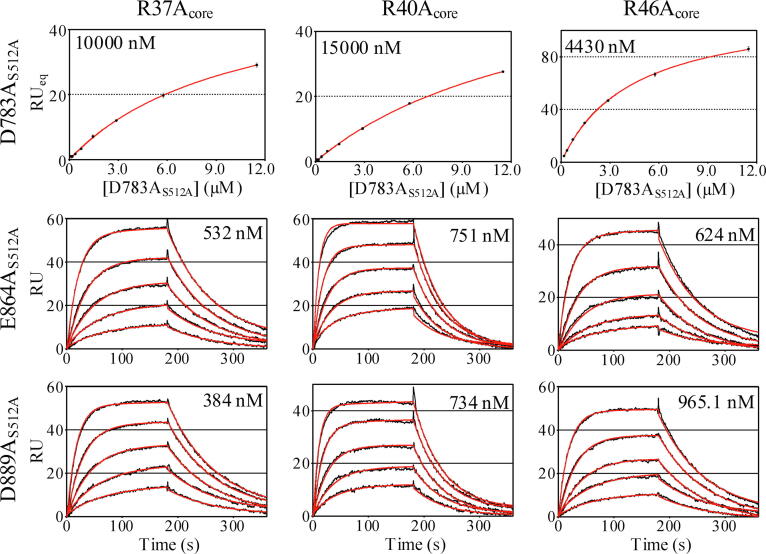# Exosite binding modulates the specificity of the immunomodulatory enzyme ScpA, a C5a inactivating bacterial protease

**DOI:** 10.1016/j.csbj.2022.08.018

**Published:** 2022-08-27

**Authors:** Monica Jain, Malgorzata Teçza, Todd F. Kagawa, Jakki C. Cooney

**Affiliations:** a Department of Biological Sciences, University of Limerick, Limerick, Ireland; b Department of Chemical Sciences, University of Limerick, Limerick, Ireland; c Bernal Institute, University of Limerick, Limerick, Ireland; d SSPC, University of Limerick, Ireland

**Keywords:** Complement factor C5a, C5a peptidase, COVID-19, exosite, Immune modulation, Substrate specificity

## Abstract

The C5a peptidase from *Streptococcus pyogenes* (ScpA) is a highly specific enzyme with potential therapeutic value. ScpA is a good model for studying determinants of specificity in the multidomain immunomodulatory enzymes (IMEs), which comprise a large family of bacterial surface proteases. The surface exposed region of ScpA has 5 main domains which includes 3 C-terminal Fn3-like domains (Fn1, Fn2 and Fn3) (Kagawa et al. 2009). Progressive deletion of the Fn3-like domains from the C-ter resulted in loss of enzyme activity and showed an important role for the Fn2 domain in enzyme function. Functional investigation of specific acidic residues on the Fn2 domain identified 3 residues 30–50 Å from the catalytic site (D783, E864 and D889) which impacted to differing degrees on binding and on catalysis, supporting the presence of an exosite on the Fn2. In particular, residue D783 was observed to impact on both substrate binding affinity and the activity of ScpA. A double mutant cycle analysis showed energetic coupling between the targeted ScpA residues and residues in the core portion (residues 1–67) of the C5a substrate. The data supports the presence of a communication network between the active site and the exosite on Fn2. These findings provide a basis for rational engineering of this important enzyme family to enhance stability, activity and/or specificity.

## Highlights

• C5a, the human complement protein is implicated in COVID-19 and sepsis.
• Proteolytic inactivation of C5a by ScpA involves interactions with a novel exosite.
• High affinity binding of C5a is shown to involve D783 of the ScpA Fn2 domain.
• Activity and affinity of ScpA is tuned by residues approx 50 Å from the active site.
• The data supports a complex dynamic process in ScpA substrate recognition.

## Graphical abstract

**Figure:**
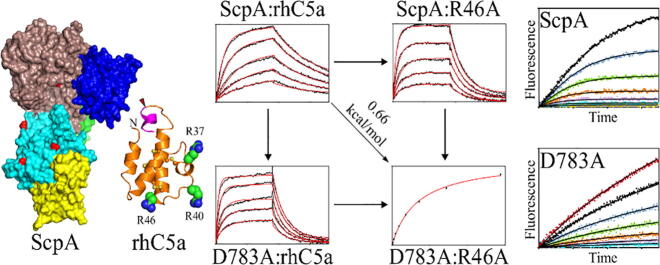

## 1 Introduction

The role of anaphylotoxins, complement split factors C5a and C3a, in complex human diseases including COVID-19 [2], [3] and sepsis [4], has sparked an interest in targeting these human proteins using biologics-based therapies. Biologics currently administered in clinical practice are predominantly monoclonal antibodies (MAbs), with anti-C5a antibodies currently in clinical trials [5]. However, key limitations of MAbs (cost and dosing levels) have resulted in an interest in the use of enzymes, including proteases, as biologics. An example of this approach is the development of MASP-3 to target complement factor C3 as a treatment for AMD (age related macular degeneration) [6]. Essential to exploitation of proteolytic enzymes as catalytic biologics is understanding how they function at a molecular level. The immunomodulatory enzyme (IMEs) ScpA, is a multi-domain, cell envelope protease (CEP) produced by the human bacterial pathogen *Streptococcus pyogenes*. With high specificity for human complement C5a and C3a [7], ScpA is thought to attenuate the host immune response to infection by proteolytic inactivation of these anaphylotoxins, making ScpA a potentially useful scaffold for engineering of therapeutic proteases. The factors governing the specific nature of the interaction of ScpA with its natural substrates are thus of crucial importance for this engineering.

ScpA is considered to be a highly selective protease, although the specificity of the enzyme has not been tested methodically. Early studies have shown that ScpA did not cleave native forms of C5, human serum albumin, ovalbumin, soybean trypsin inhibitor, carbonic anhydrase, α-lactalbumin, myosin, and cytochrome *c* [8]. In addition, the highly homologous ScpB enzyme inactivated bovine C5a but not the mouse or rat proteins [9]. Recent studies with high concentrations of substrate and enzyme (5 mM C3a or C5a and 0.5 µM ScpA) indicate that ScpA cleaves C3a more efficiently than C5a [7]. As with hC5a, ScpA inactivates hC3a by releasing the seven C-terminal tail residues from the core portion of the anaphylotoxin. Significantly, we have recently established binding enzyme kinetic parameters for the enzyme using surface plasmon resonance (SPR) and a FRET-labelled C5a enzyme assay respectively. For the first time, this indicated the enzyme was capable of cleaving C5a at the physiological concentrations of C5a found in infections with a *K*_D_ of 35 nM and *K*_m_ of 185 nM [10].

**Fig. 1:**
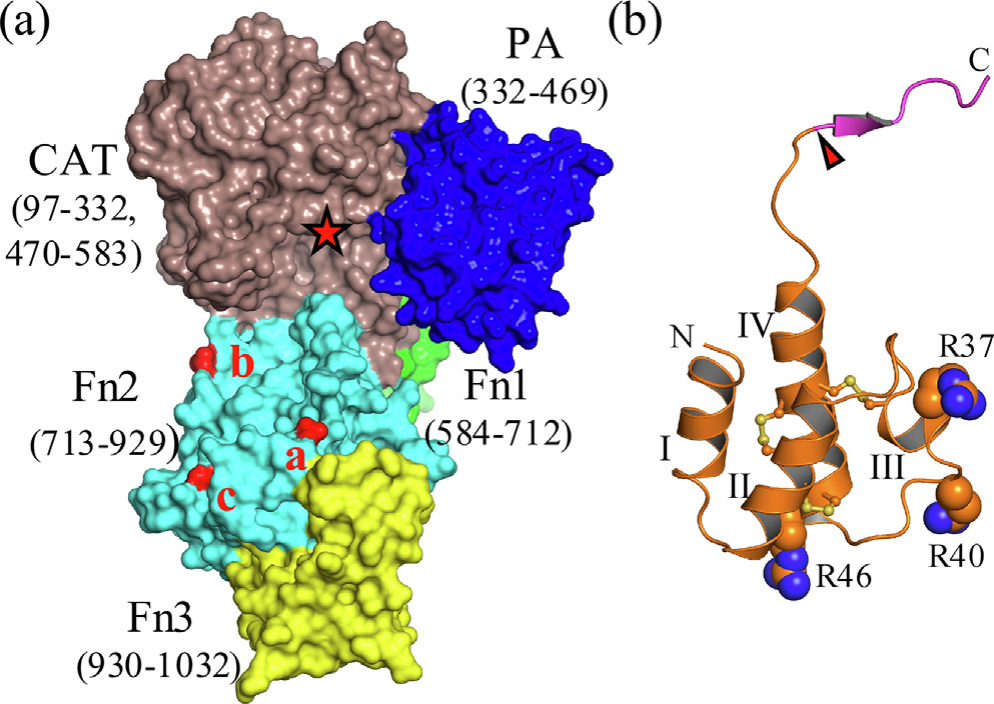
Structure of ScpA and C5a. Panel (a) Surface rendering of ScpA. The domains are coloured according to Kagawa et al. [1]. The numbers in parenthesis are the residues in each domain. The approximate location of the catalytic site is marked with a red star. Residues mutated in ScpA for this study are indicated by red surface rendering. D783 (labelled ‘a’) is located near the interface with the Fn3 domain and 48 Å from the catalytic serine (S512). E864 (labelled ‘b’) is at the outer edge of the Fn2 domain, 30 Å from S512 near the entrance to the active site. D889 (labelled ‘c’) is also positioned at the outer edge of the Fn2 domain 46 Å from S512, adjacent to the Ca^2+^ binding site. Panel (b) Cartoon diagram showing location of mutations in hC5a. Scissile bond between H67 and K68 is indicated with a red arrowhead. The ‘tail’ is coloured magenta. The C5a ‘core’ region (residues 1–67) is coloured orange and the C-terminal ‘tail’ (residues 68–74) magenta. Mutated C5a residues (R37, R40 and R46) are rendered as spheres. The main helices are labelled I-IV with disulfide bridges shown as yellow ball and stick models. (For interpretation of the references to colour in this figure legend, the reader is referred to the web version of this article.)

The crystal structure of the active ScpA enzyme (Fig. 1a) revealed a subtilisin-like catalytic domain structurally augmented by insertion of a PA domain, followed by 3 tandemly arranged C-terminal fibronectin type III domains (Fn1–Fn3) [1]. The spatial juxtaposition of the PA domain and the Fn2 domain over the prime and non-prime side of the active site cleft, respectively, occludes the active site, hindering access to the catalytic machinery by larger substrates. The reported model of the ScpA-hC5a enzyme-substrate (ES) complex suggested that the PA and Fn2 domains could participate in substrate interactions in the active site and at an exosite, respectively. Further support for the role of the PA domain and Fn domains in substrate selectivity in CEPs was demonstrated for the PrtPs of *Lactococcus lactis* [11], [12] and more recently for the PA domain of ScpC (SpyCEP) of *Streptococcus pyogenes* [13].

In the model of the ScpA-hC5a complex [1], positioning of the scissile bond in C5a (H67-K68) near the catalytic site required that the hC5a residues 65–74 adopt an extended conformation (Fig. 1b). Interactions with the bulky C5a_core_ (residues 1–67 of C5a, also referred to as the *N*-terminal product of proteolysis P_N_) occur outside the active site with residues of the Fn2 domain, and these interactions are predicted to make the largest contribution to substrate binding affinity and to involve a significant contact surface area stabilized by a series of ionic interactions. The model also proposed that ionic interactions with residues K68, D69 and R74 in the C5a C-ter tail would be involved in substrate binding.

Further studies on the impact of different regions of CEPs have focussed on the Streptococcal enzymes ScpA and ScpC (SpyCEP). Structure solution of ScpC and derivatives confirmed a similar organization of the catalytic to Fn3 domains to ScpA and described additional C-ter domains which were proposed to contribute to substrate specificity [14]. Molecular dynamics studies of ScpC indicate a degree of motion in the PA domain of the enzyme and the proposal that this would gate access to the active site in some manner. In addition, McKenna et al. [13] proposed that an interaction between substrates and the PA domain could perpetuate allosteric events resulting in conformation changes in the body of the enzyme which are required for activity. Binding of substrate (IL-8) to ScpC was investigated using SPR and a *N*-ter region comprising domains up to and including the Fn1 domain bound substrate with high affinity (*K*_D_ 13.1 nM), whilst loss of the Fn1 resulted in reduced affinity (*K*_D_ 927 nM) [15]. Other modes of analysis presented indicated a low affinity binding to the regions distal to the Fn1 domain. These studies point to roles for the non-catalytic domain in substrate specificity and catalysis and further develop observations made for PrtP.

Recent studies on the biochemical properties of ScpA indicated that the enzyme bound C5a with high affinity, *K*_D_ 34 nM [10]. Furthermore, the C5a_core_ contribute 89% of ΔG°_bind_, based on a *K*_D_ of 240 nM, implying an interaction with the enzyme outside the active site cleft. The recruitment of C5a to the ScpA surface was shown to be significantly impacted upon by environmental ionic strength. The importance of electrostatics in substrate recruitment was supported by additional studies on C5a where point mutations in 4 R residues (R37, R40, R46 and R74) significantly decreased binding affinity as measured by surface plasmon resonance (SPR). R37, R40 and R46 are located in the core of C5a, more than 20 Å from the scissile bond. Thus, the mutagenesis experiments further support the participation of exosite-type interactions in substrate binding. While acknowledging the likely role of the PA domain in the mode of action of ScpA, the current study focusses on investigating the presence of an exosite on the Fn2 domain. Using a combination of domain deletions followed by targeted point mutations in ScpA and double mutant cycle analysis on the ScpA-C5a system, we show that the Fn2 has a key role in enzyme activity and substrate binding. Residues in the Fn2 of ScpA were shown to contribute to the interaction with C5a, and pairs of interacting residues (one enzyme, one substrate) were identified which will guide engineering of the enzyme and further modelling of the ES complex.

## 2 Materials and methods

### 2.1 Preparation of recombinant proteins.

The cloning and production of recombinant ScpA, ScpA_S512A_ and human C5a (rhC5a) and derivatives have been described previously [1]. For the purposes of this work ScpA comprises amino acid residues 31–1032 of the translated sequence unless otherwise indicated.

**Supplementary figure 1:**
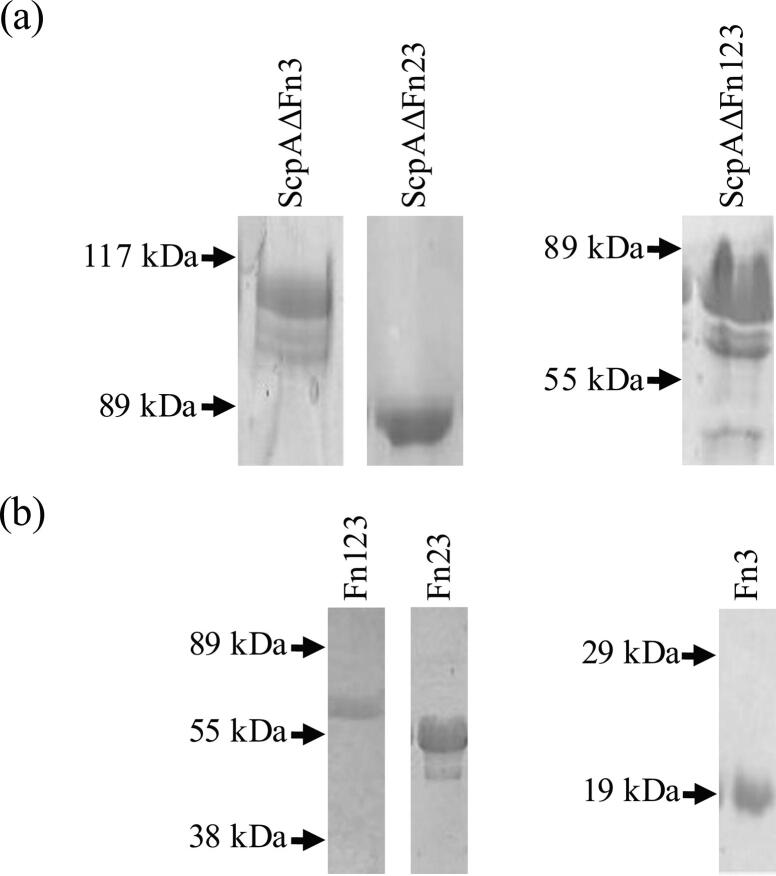


**Supplementary figure 2:**
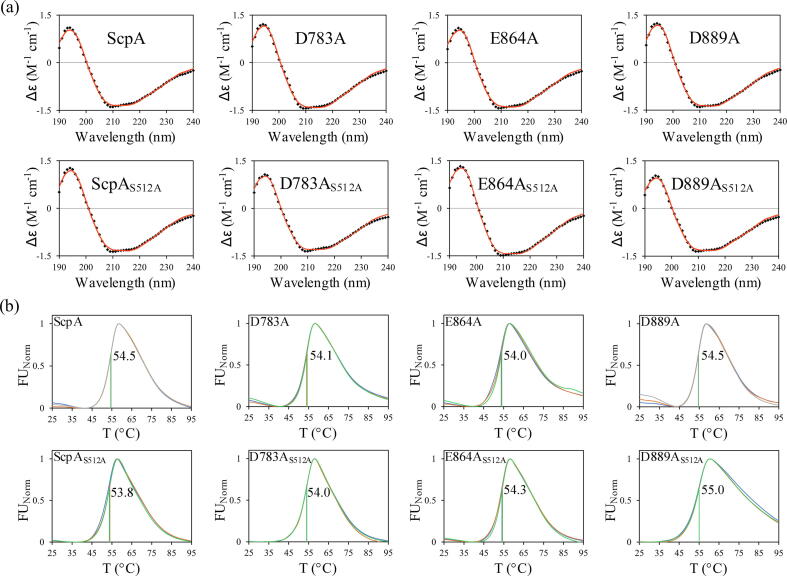


The plasmid expressing ScpA (pGEX ScpA_(31-1032)_) was used as a template to generate domain drop-out mutations (DDOs) (Fig. S1 and Table 1) by standard PCR methods. The same template was used to generate plasmids expressing ScpA_D783A_, ScpA_E864A_ and ScpA_D889A_ using the QuikChange II site directed mutagenesis kit (Stratagene, USA). For simplicity in the text these will be called D783A, E864A and D889A, respectively. Generation of the active site serine to alanine (S512A) mutation of all constructs for use in SPR studies also employed the QuikChange II system, generating ScpA_S512A,D783A_, ScpA_S512A,E864A_ and ScpA_S512A,D889A_. For simplicity in the text these will be called D783A_S512A_, E864A_S512A_ and D889A_S512A_, respectively. The proper folding of ScpA_D783A_ was confirmed by X-ray crystallography (Data in Brief), and the coordinates and structure factors deposited at the Protein Data Bank (PDB 7YZX). In addition, the crystal structures of ScpA [1] and ScpA_S512A_ [10] have been solved previously. The structures are nearly identical based on RMSD of Cα atoms. The fold of the remaining ScpA proteins were confirmed with CD spectropolarimetry to assess secondary structure and differential scanning fluorimetry to measure melting temperatures (T_m_). All ScpA forms were observed to have similar T_m_s and CD spectra (Fig. S2 and Table S1). Additional details for the constructions, production, purification and evaluation of recombinant protein are provided in Supplemental Information (Sections SI1 and SI2).

**Table 1:** Domain dropout characterization.

Construct tested	Schematic^a^	C5a-ase	MS^b^	Scissile bond
ScpA		+	10883	H67-K68
ScpAΔFn3		+	10885	H67-K68
ScpAΔFn23		–	ND	ND
ScpAΔFn123		–	ND	ND
Fn123		–	ND	ND
Fn23		–	ND	ND
ScpAΔFn23 + Fn123		+	9783	V57-A58
ScpAΔFn23 + Fn23		+	9787	V57-A58
ScpAΔFn123 + Fn123		+	9785/10883	V57-A58/H67-K68
ScpAΔFn123 + Fn23		–	ND	ND

a Domain coloring described in Fig. 1.

b Mass spectrometry of products when hydrolysis observed. The observed mass is associated with the larger N-terminal product of hydrolysis.

Recombinant human C5a (rhC5a) and its R37A, R40A and R46A mutants were produced as *N*-ter *hexa*-histidine tagged (HT) fusion proteins as previously described [10]. Generation and purification of the larger *N*-terminal cleavage products of ScpA inactivation (rhC5a_core_) and the fluorescently labelled form of rhC5a (rhC5a_C75_-BODIPY) are also described in Teçza et al. [10]. As for ScpA and its mutants, the names of rhC5a forms have been simplified in the text. The full -length forms of the rhC5a mutants (rhC5a_R37A_, rhC5a_R40A_ and rhC5a_R46A_) will be referred to as R37A, R40A and R46A. The inactive cleaved forms of the rhC5a mutants will be referred to as R37A_core_, R40A_core_, and R46A_core_. Mass spectrometry was used to confirm the scissile bond in all rhC5a cleavage products and for assessing the extent of rhC5a_C75_-BODIPY labelling [10]. Intact mass analysis of rhC5a and rhC5a_C75_ proteins were performed in-house on a Bruker UltrafleXtreme instrument (Bruker Daltonik GmbH, Germany), using Compass 1.4 software as previously described [10].

### 2.2 ScpA activity assays.

#### 2.2.1 End-point activity assay

The activity of recombinant ScpA proteins against rhC5a, was examined in cleavage assays containing 18 μM C5a peptide and 5 nM ScpA in PBS. The reactions were incubated at 37 °C for 20 min and analyzed by SDS-PAGE and MS. End point assays for domain drop-out mutations (DDOs) of ScpA were performed and the mass of the predominant products determined by MS (Table 1). These assays contained high concentration of enzyme (110 nM) and substrate (20 µM) and were incubated for 6 hr. The results were analysed by SDS-PAGE [16]. Additional gel assay methods are described in Supplement Information SI2.

#### 2.2.2 Enzyme kinetics assays

**Supplementary figure 3:**
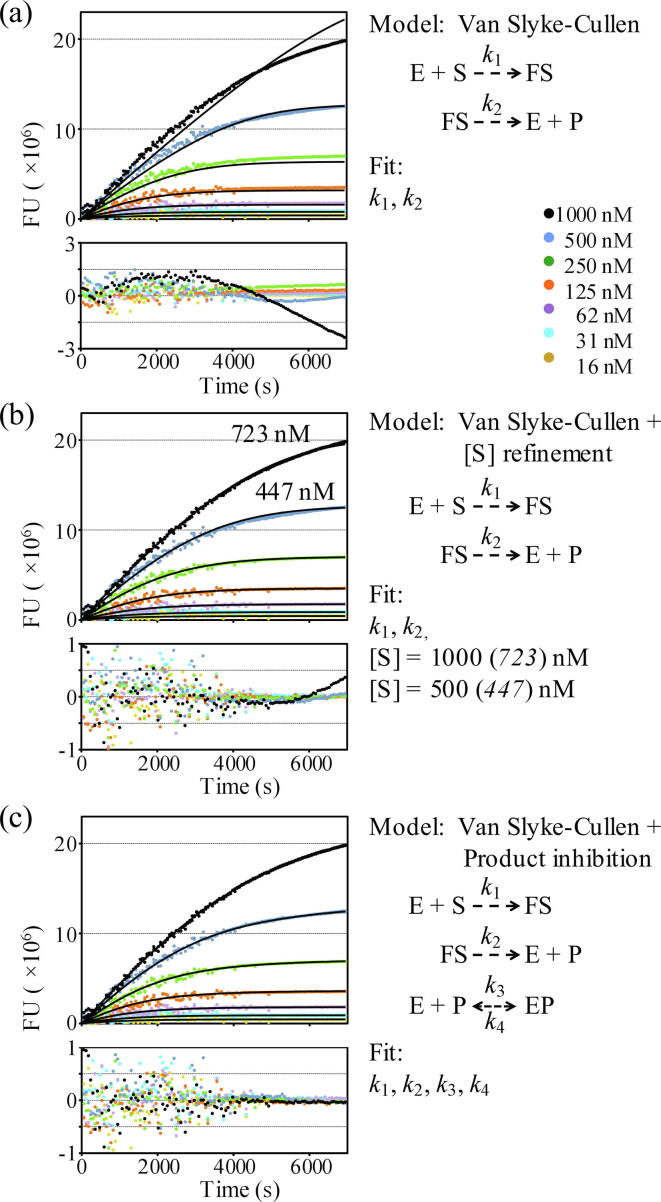


Enzyme kinetics assay were performed as previously described [10]. Briefly, evolution of fluorescence was measured at a fixed concentration (0.2 nM) of ScpA_31-1032_, D783A, E864A and D889A with rhC5a_C75_-BODIPY substrate in 1× PBS with 0.1% (v/v) Tween 20. Measurements were made on a Berthold LB941 fluorescence plate reader (Berthold Technologies, UK) with excitation at 485 nm and emission at 520 nm and a 2 s data acquisition time for 8000 s. Kinetic parameters for ScpA, E864A and D889A were determined with substrate concentrations ranging between 15.6 and 1000 nM while 15.6 to 2000 nM substrate was used for the D783A enzyme. In all cases the enzyme concentration was much less than the substrate concentration. Experiments were performed at 20 °C, in quadruplicate. Following correction for spontaneous substrate hydrolysis the progress curves were analysed using the DYNAFIT software package [17]. Progress curves initially fit with the minimal Van Slyke-Cullen mechanism [18] were found to have systematic residuals at higher substrate concentrations indicating product inhibition during the course of the experiment (see Fig. S3). Addition of product inhibition equilibrium to the mechanism effectively eliminated the residual signal and allowed determination of steady state enzyme kinetic parameters (*K*_m_ and *k*_cat_).

### 2.3 Surface plasmon resonance studies.

SPR binding parameters have been previously published for ScpA binding to rhC5a, rhC5a_core_, R37A, R40A and R46A [10]. To maintain consistency for comparisons with other interactions in this study, these interactions were re-evaluated.

All SPR data were measured at 25 °C with a BIAcore X100 system (GE Healthcare, UK) in Hepes buffer (10 mM Hepes-KOH pH 7.4, 150 mM NaCl, 0.005% (v/v) Tween 20, 50 μM EDTA). Data was obtained for binding of the full-length and core forms of the His-tagged C5a peptides R37A, R40A and R46A ligands to the D783A_S512A_, E864A_S512A_, D889A_S512A_ ligates. Interactions were assessed using an NTA sensor chip (GE Healthcare, UK) with approximately 20–25 response units (RU) of immobilized HT ligand using a flow rate of 30 μL/min. For each binding pair, the top concentration of ligate used are reported on Table S2. Association and dissociation phases were each monitored for 200 s. Double referencing was used to remove the effects associated with buffer changes. In addition, the signal associated with non-specific interactions with the chip surface was subtracted from sensorgrams when ligate concentrations greater than 180 nM were used. Unless otherwise stated the sensorgrams were fit in a global analysis with the BIAevaluation 4.1 curve fitting software using 1:1 Langmuir model with a drifting baseline (GE Healthcare, UK). Assays examining the interaction of full-length and core forms of C5a R37A, R40A and R46A with D783A_S512A_ required fitting of the response units at equilibrium to obtain a *K*_D_ due to the very fast dissociation rates between these binding pairs. The experiments for each binding pair were conducted in triplicate. Values of binding constants reported on Table 2, Table 3 represent the mean and standard deviation from three experiments. The binding energies (ΔG°_bind_) were calculated with Eq. (1):

(1) $$\Delta \text{G}{{}^{\circ}}_{\text{bind}}=\text{RTln}\left[K_{\text{D}}\right]$$

where R is the gas constant (1.986 cal mol^−1^ K^−1^) and T is the temperature in Kelvin (T = 298 K). Binding energies were used in a double mutant cycle (DMC) analysis to assess the additivity and thus energetic coupling of mutant pairs in the ligand and ligate. Coupling energies (ΔΔΔG_C_) were calculated as described by Mesrouze et al. [19] with Eq. (2):

(2) $$\begin{array}{c} \Delta \Delta \Delta {\text{G}}_{\text{C}}=\Delta \text{G}{{}^{\circ}}_{\text{bind}}\left(\text{ScpA}:\text{rhC5a}\right)+\Delta \text{G}{{}^{\circ}}_{\text{bind}}\left({\text{ScpA}}_{\text{mut}}:{\text{rhC5a}}_{\text{mut}}\right)\\ \;-\;\Delta \text{G}{{}^{\circ}}_{\text{bind}}\left(\text{ScpA}:{\text{rhC5a}}_{\text{mut}}\right)\;-\;\Delta \text{G}{{}^{\circ}}_{\text{bind}}\left({\text{ScpA}}_{\text{mut}}:\text{rhC5a}\right) \end{array}$$

where the coupling energy for a mutant pair is dependent on the binding energies measured for the wild-type interaction (ScpA:rhC5a), the interaction between the two mutant forms (ScpA_mut_:rhC5a_mut_) as well as the interaction between wild-type and mutant forms of both the ligand and ligate (ScpA:rhC5a_mut_ and ScpA_mut_:rhC5a respectively).

**Table 2:** Kinetic and thermodynamic parameters for binding to full length rhC5a and its mutants.

Enzyme:substrate	*k*_a_ × 10^4^ (M^−1^ s^−1^)^a^	*k*_d_ × 10^−3^ (s^−1^)^a^	*K*_D_ (nM)^a^	ΔG°_bind_ (kcal/mol)^b^
ScpA_S512A_:rhC5a	20.9 ± 2.4	7.0 ± 0.4	34 ± 4	−10.18
ScpA_S512A_:R37A	8.12 ± 0.49	17.6 ± 0.2	217 ± 11	−9.081
ScpA_S512A_:R40A	9.44 ± 0.07	27.4 ± 0.5	290 ± 3	−8.909
ScpA_S512A_:R46A	10.3 ± 0.3	22.6 ± 0.3	220 ± 4	−9.073
D783A_S512A_:rhC5a	7.54 ± 0.05	23.7 ± 0.1	314 ± 2	−8.862
D783A_S512A_:R37A	ND	ND	2000 ± 100^c^	−7.78
D783A_S512A_:R40A	ND	ND	1500 ± 100^c^	−7.93
D783A_S512A_:R46A	ND	ND	669 ± 23^c^	−8.414
E864A_S512A_:rhC5a	26.0 ± 1.9	6.4 ± 0.2	25 ± 3	−10.37
E864A_S512A_:R37A	15.0 ± 0.2	10.09 ± 0.13	67.1 ± 1.9	−9.775
E864A_S512A_:R40A	7.85 ± 0.11	27.1 ± 0.3	346 ± 5	−8.805
E864A_S512A_:R46A	34.4 ± 1.3	21.3 ± 0.1	62.1 ± 2.6	−9.821
D889A_S512A_:rhC5a	26.3 ± 0.2	7.0 ± 0.5	27 ± 2	−10.32
D889A_S512A_:R37A	13.68 ± 0.27	9.34 ± 0.18	68.3 ± 2.6	−9.765
D889A_S512A_:R40A	27.3 ± 1.6	25.9 ± 0.6	94.9 ± 3.6	−9.570
D889A_S512A_:R46A	14.85 ± 0.34	12.85 ± 0.17	86.57 ± 2.92	−9.6245

a Reported as mean and standard deviation from 3 experiments.

b ΔG°_bind_ = RTln[*K*_D_], R = 1.986 (cal mol^−1^ K^−1^), T = 298 K.

c *K*_D_ values ± SE obtained from equilibrium binding analysis of data from 3 experiments.

**Table 3:** Kinetic and thermodynamic parameters for binding to full length rhC5a_core_ and its mutants.

Enzyme: product	*k*_a_ × 10^4^ (M^−1^ s^−1^)^a^	*k*_d_ × 10^−3^ (s^−1^)^a^	*K*_D_ (nM)^a^	ΔG°_bind_ (kcal/mol)^b^
ScpA_S512A_:rhC5a_core_	4.50 ± 0.11	7.5 ± 0.2	170 ± 10	−9.23
ScpA_S512A_:R37A_core_	3.33 ± 0.02	15.10 ± 0.13	453 ± 5	−8.645
ScpA_S512A_:R40A_core_	1.93 ± 0.10	25.8 ± 0.3	1340 ± 70	−8.004
ScpA_S512A_:R46A_core_	2.20 ± 0.05	15.21 ± 0.41	691 ± 4	−8.395
D783A_S512A_:rhC5a_core_	1.097 ± 0.031	21.6 ± 0.5	1970 ± 80	−7.775
D783A_S512A_:R37A_core_	ND	ND	10000 ± 1000^c^^,^^d^	−6.80
D783A_S512A_:R40A_core_	ND	ND	15000 ± 1000^c^^,^^d^	−6.57
D783A_S512A_:R46A_core_	ND	ND	4330 ± 110^c^	−7.309
E864A_S512A_:rhC5a_core_	4.93 ± 0.06	4.38 ± 0.04	88.7 ± 0.6	−9.610
E864A_S512A_:R37A_core_	2.28 ± 0.08	12.1 ± 0.2	532 ± 19	−8.550
E864A_S512A_:R40A_core_	2.61 ± 0.11	19.5 ± 0.1	751 ± 26	−8.346
E864A_S512A_:R46A_core_	2.12 ± 0.05	13.2 ± 0.1	624 ± 11	−8.456
D889A_S512A_:rhC5a_core_	4.44 ± 0.02	4.0 ± 0.1	90 ± 3	−9.60
D889A_S512A_:R37A_core_	3.01 ± 0.03	11.54 ± 0.17	384 ± 6	−8.743
D889A_S512A_:R40A_core_	2.48 ± 0.04	18.2 ± 0.5	734 ± 32	−8.359
D889A_S512A_:R46A_core_	1.254 ± 0.062	12.08 ± 0.07	965.1 ± 54.0	−8.1974

a Reported as mean and standard deviation from 3 experiments.

b ΔG°_bind_ = RTln[K_D_], R = 1.986 (cal/mol K^−1^), T = 298 K.

c *K*_D_ values ± SE obtained from equilibrium binding analysis of data from 3 experiments.

d *K*_D_ values are lowest estimates limited by the highest ligate concentration of 11,500 nM used in the analysis.

## 3 Results

### 3.1 Deletion of the fibronectin type III domains in ScpA impacts on stability and activity of the enzyme.

Modeling of the ScpA:C5a complex [1], and mutagenesis studies on related CEPs from *L. lactis* posit the significance of residues in the Fn2 domain in substrate interactions [11], [12]. To examine the role of the C-terminal Fibronectin type III domains in ScpA, the Fn1, Fn2 and Fn3 domains (see Fig. 1a) were sequentially deleted, and the activity of these mutants tested. The constructs are labelled ScpAΔFn3, ScpAΔFn23 and ScpAΔFn123, indicating the deletion of the Fn3, Fn2-Fn3 and Fn1-Fn3 domains, respectively. Diagrams and nomenclature for these constructs are shown in Table 1. Deletion of either the Fn2-Fn3 (ScpAΔFn23) or Fn1-Fn3 (ScpAΔFn123) domains resulted in expression of a stable protein while ScpAΔFn3 was marginally unstable (Fig. S1a).

In end-point activity assays, C5a-ase activity was not observed for constructs ScpAΔFn123 and ScpAΔFn23 whereas activity was observed for ScpAΔFn3. Thus, only the deletion mutant with an Fn2 domain retains C5a-ase activity albeit at approximately 13% the activity of the wild-type enzyme (data not shown). Mass spectrometry (MS) of the resultant larger product showed that cleavage occurs between residue H67 and K68 as observed for the wild-type enzyme (Table 1). The inability of the ScpAΔFn123 and ScpAΔFn23 mutants to cleave the substrate also implies that the catalytic domain with the inserted PA domain alone is not capable of capturing the substrate in a productive manner.

While the activity of ScpAΔFn123, which comprises only the catalytic domain and PA domain, was not restored by addition of Fn23, activity was restored by addition of Fn123. MS analysis of the reaction identified a mixture of products cleaved between V57-A58 and at the expected cleavage site between H67-K68. This indicates that ScpAΔFn123 retains its ability to hydrolyze substrate but does not bind the substrate appropriately in the absence of the C-terminal domains. The activity of ScpAΔFn23 was restored by addition of either Fn123 or Fn23. Analysis of the products in both ScpAΔFn23 complementation experiments showed that cleavage occurs between V57 and A58. The activity of ScpAΔFn23 when combined with Fn23 was 1.3% and 1% when complimented with Fn123 (data not shown).

Taken together these studies suggested an essential role in substrate binding for the Fn2 domain. Therefore, specific point mutations were introduced to probe the role of the Fn2 domain in C5a inactivation.

### 3.2 Activity of the D783A mutant is distinct from the wild-type, E864A and D889A forms of ScpA

Fn2 residues D783, E864 identified previous [1] and residue D889 identified by additional modelling (Kagawa and Cooney, unpublished data) were selected for point mutation to further investigate the role of the exosite in ScpA.

**Fig. 2:**
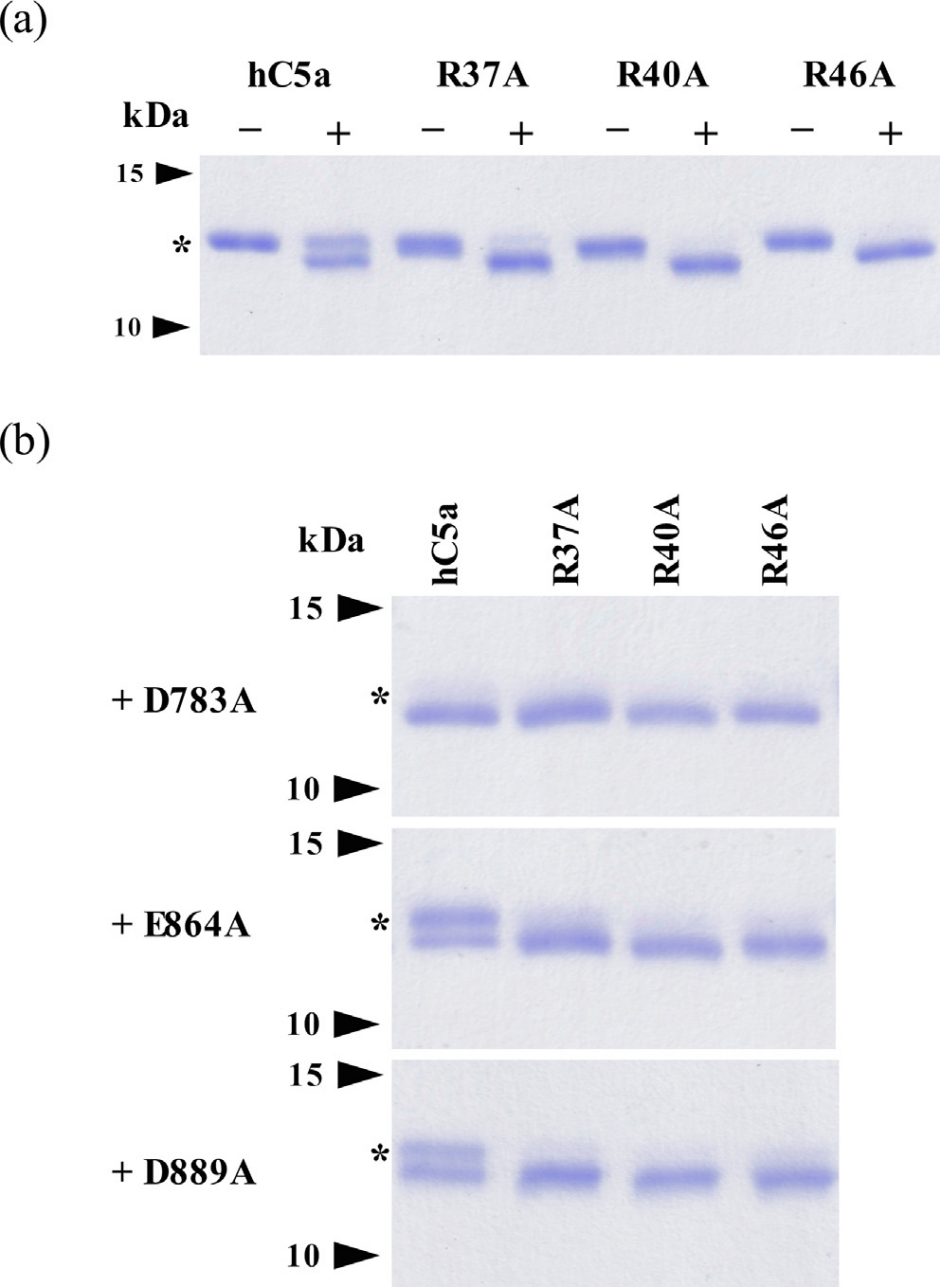
End-point digestion of C5a by ScpA. Panel (a) SDS-PAGE analysis of ScpA cleavage of rhC5a and rhC5a mutants. Panel (b) SDS-PAGE analysis of ScpA mutants’ ability to cleave rhC5a and rhC5a mutants. The activity of the wild-type ScpA was assessed in a gel-based assay with 5 nM enzyme and 18 µM rhC5a substrate. Activity of the ScpA mutant forms against rhC5a as well as the R37A, R40A and R46A mutants of rhC5a were similarly examined. Assays were conducted at 37 °C for 20 min and products were analyzed by Mass Spectrometry to confirm cleavage sites. The asterisks in both panels indicate the location of bands associated with un-cleaved rhC5a and rhC5a mutants.

**Supplementary figure 4:**
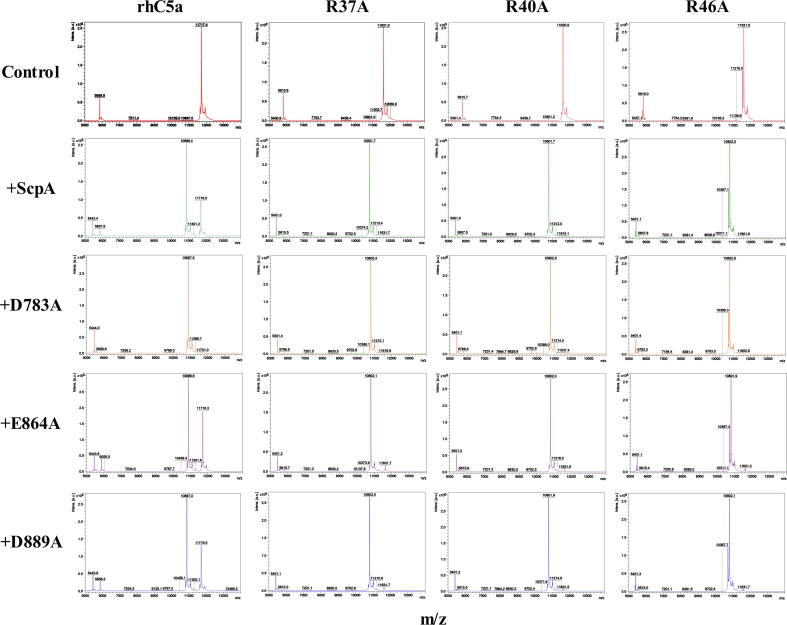


Following purification, the activity of the ScpA point mutants (D783A, E864A and D889A) were assessed by end-point gel assay. The activity of ScpA (Fig. 2a) and its mutant forms (Fig. 2b) were examined against rhC5a as well as the R37A, R40A and R46A mutants. Under the conditions used in the assay, ScpA was observed to cleave all forms of C5a tested (Fig. 2a). Similarly, all mutant forms of ScpA examined retain the ability to cleave C5a and the C5a mutants (Fig. 2b). Mass spectrometry of reaction products confirmed that cleavage occurs between H67 and K68 in all reactions (Supplement Fig. S4 and Table S3). This suggests that the mutations in ScpA and rhC5a did not alter the ability of the enzymes to properly orient the substrate in the active site.

Interestingly, the assays exposed differences in the abilities of the ScpA mutants to cleave rhC5a. The D783A mutant (Fig. 2b) hydrolysed rhC5a to a greater extent than the wild-type (Fig. 2a), E864A and D889A forms of ScpA (Fig. 2b). In addition, the wild-type, E864A and D889A forms cleave rhC5a less efficiently than the R37A, R40A or R46A mutants of the substrate. The decreased efficiency in processing rhC5a, as compared to the mutants, was not observed for ScpA in the previously published assay [10]. It is possible that the use of an 8-fold lower concentration of enzyme in the current study (5 nM vs 40 nM) was necessary to reveal differences in substrate cleavage rates in these end point assays. To better understand the basis for the difference in C5a-ase activity, interactions with the substrate and product were examined for all ScpA forms with SPR.

### 3.3 D783 in Fn2 domain contributes to stability of the enzyme-substrate complex

**Fig. 3:**
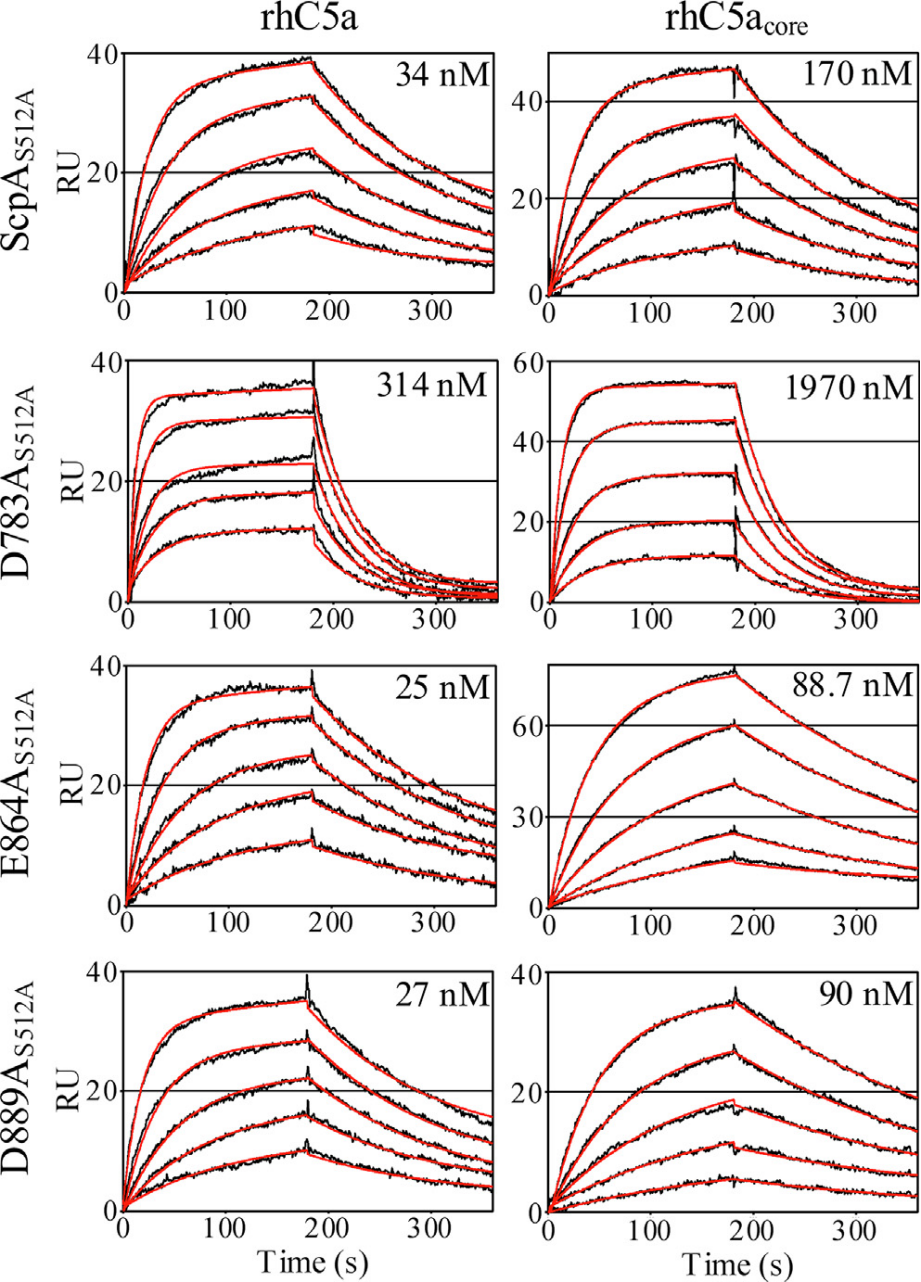
SPR analyses of binding to full length rhC5a and rhC5acore. Representative SPR sensorgrams of ScpAS512A and mutant derivatives binding to immobilized full-length rhC5a and rhC5acore. Observed data (black lines) are shown with curves obtained from global fitting of data with a 1:1 Langmuir model for binding (red lines). The mean K_D_ value obtained from 3 experiments is reported in the upper right-hand corner of the respective panel. (For interpretation of the references to colour in this figure legend, the reader is referred to the web version of this article.)

Sensorgrams for binding of all ScpA forms to the full-length rhC5a substrate are shown in Fig. 3 and binding parameters reported on Table 2. The *K*_D_ for rhC5a binding to ScpA_S512A_ is 34 nM, in agreement with the earlier study by Teçza et al. [10]. The *k*_a_ and *k*_d_ values in the current study are 20.9 × 10^4^ M^−1^ s^−1^ and 7.0 × 10^−3^ s^−1^ respectively (Table 2), both approximately 1.5-fold higher than previously observed. The discrepancy in values is potentially related to higher concentrations of ScpA_S512A_ used in the current study, variations across individual chips or sample preparations used in each experiment. To allow for better comparisons with the ScpA mutants, the current values of the ScpA binding parameters will be used in this study.

The *K*_D_ for the interaction between the D783A_S512A_ mutant and rhC5a is 314 nM, more than 9-fold higher than observed for ScpA_S512A_. The increase in *K*_D_ for the D783A mutant results from 2.8-fold slower association rate and 3.3-fold faster dissociation rate, indicating that D783 is involved in the formation of enzyme-substrate complex as well as contributes stabilizing interactions in the bound complex. This single point mutation in Fn2 domain decreased binding affinity (ΔΔG°_bind_) by 1.32 kcal/mol and supports the participation of the Fn2 domain in substrate recognition.

In contrast to the D783, E864 and D889 do not contribute significantly to the stability of the enzyme:substrate complex. The binding affinity of E864A_S512A_ and D889A_S512A_ for the rhC5a substrate are in the low nM range similar to that observed for ScpA_S512A_. The ΔΔG°_bind_ for the E864A and D889A mutants are 0.20 and 0.14 kcal/mol respectively. The small decrease in *K*_D_ results mainly from a marginally faster association rate for both mutant forms. The dissociation rates for substrate binding are nearly identical to the *k*_d_ for ScpA_S512A_.

### 3.4 Binding of D783A, E864A and D889A mutants to the core portion of C5a

Previously, ScpA_S512A_ was observed to bind the larger *N*-terminal cleavage product of C5a (residues 1–67), referred to as the C5a core (rhC5a_core_ or P_N_), with nM affinity (*K*_D_ 240 nM) accounting for 89% of the binding energy for the full-length substrate [10]. Binding of rhC5a_core_ to the ScpA mutants were examined to assess whether the product binds in a similar manner to these forms of ScpA (Fig. 3 and Table 3).

The *K*_D_ for rhC5a_core_ binding to ScpA_S512A_ in the current study is 170 nM, in agreement with our previously published studies [10], with *k*_a_ and *k*_d_ values of 4.50 × 10^4^ M^−1^ s^−1^ and 7.5 × 10^−3^ s^−1^, respectively (Table 3). The D783A_S512A_, E864A_S512A_ and D889A_S512A_ mutants bind rhC5a_core_ with *K*_D_s of 1970, 88.7 and 90 nM, respectively. The lower affinity of D783A_S512A_ for the product as compared to ScpA_S512A_, E864A_S512A_ and D889A_S512A_ mirrors the trend observed for binding of the substrate (Section 3.3). The associated rhC5a_core_ binding energies are −7.8 kcal/mol for D783A_S512A_ and −9.6 kcal/mol for both E864A_S512A_ and D889A_S512A_. Thus, as for ScpA_S512A_, interactions with the core portion of C5a accounts for the majority (88, 92 and 93%) of substrate binding energy in the ScpA mutants.

As with ScpA, the decrease in binding affinity for the P_N_ product is predominantly due to a slower association rate. Relative to substrate binding, the *k*_a_ increases by 6.9-, 5.3- and 5.9-fold for D783A_S512A_, E864A_S512A_ and D889A_S512A_ respectively as compared to 4.6-fold for ScpA_S512_. The enhanced association rates in the presence of the tail (*i.e.* in substrate binding) in these studies support a previous suggestion [10] that interactions with the tail residues are potentially involved in sensing and communicating the status of prime region of active site to other regions involved in productive binding of the substrate in the catalytic cycle (*i.e.* the Fn2 domain).

Taken together, the SPR studies on rhC5a and rhC5acore, support that the substrate and product binds to all mutants in the same manner as the wild-type ScpA with significant interactions with residues in the core of C5a.

### 3.5 Residue D783 impacts on ScpA catalytic turnover and alleviates product inhibition

Enzyme kinetic studies were conducted with a fluorescently labelled full-length C5a substrate as described previously [10]. The Van Slyke-Cullen mechanism was modified to account for product inhibition observed at higher substrate concentrations (Fig. S3).

The enzyme kinetic parameters for ScpA (*k*_cat_ = 1.006 s^−1^ and *K*_m_ = 189 nM) are in good agreement with previously published values obtained without considering product inhibition (*k*_cat_ = 0.886 s^−1^ and *K*_m_ = 185 nM). No substrate inhibition was observed with substrate concentrations up to 500 nM [10]. However, the higher substrate concentrations used in this study exposed significant product inhibition during the reaction which required modification of the mechanism to better account for the observed data. Product inhibition was also observed with D783A, E864A and D889A mutants, thus the progress curves were fit also using this strategy.

**Fig. 4:**
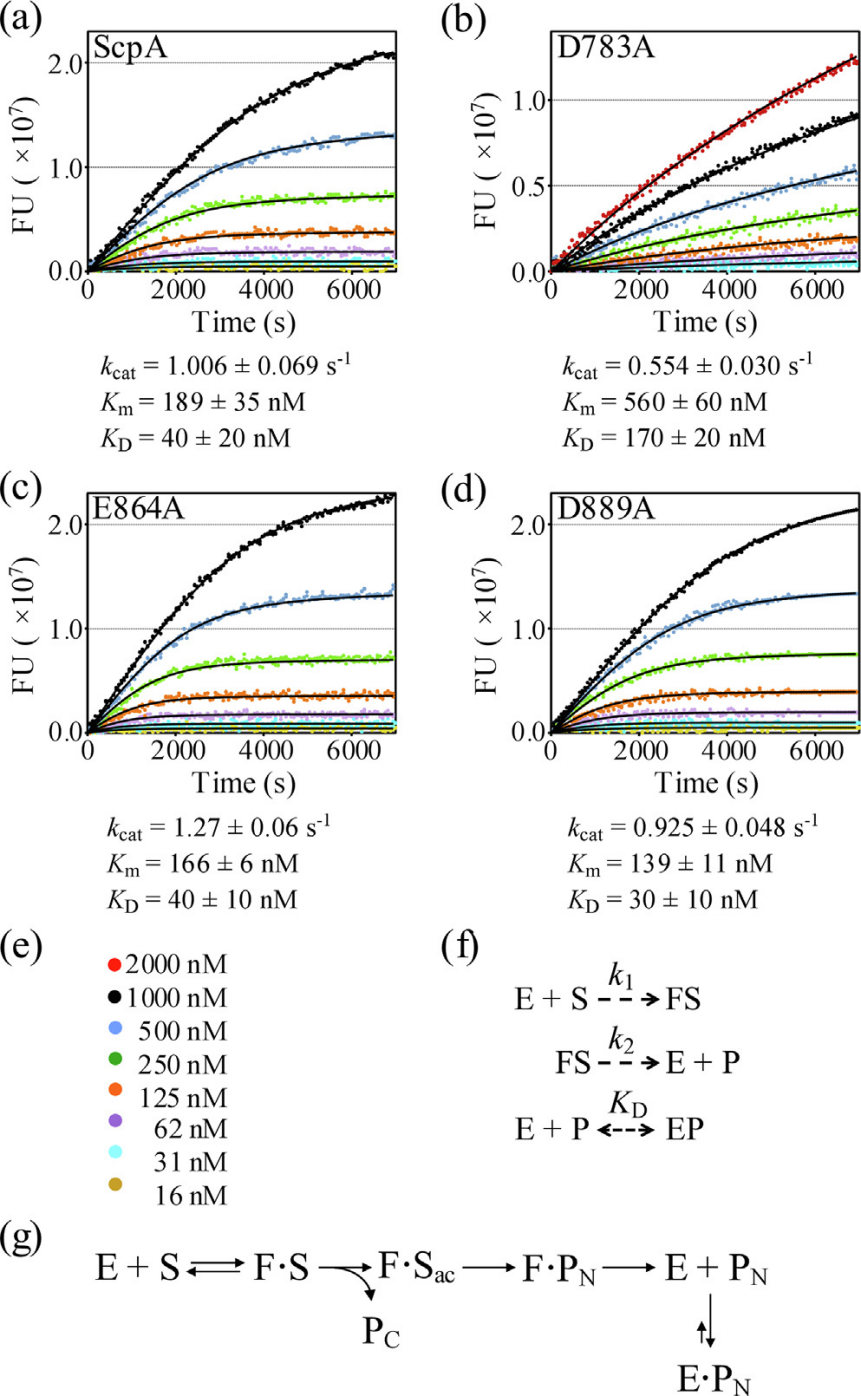
Enzyme kinetic data for ScpA and ScpA mutants. Panels (a) through (d) progress curves for cleavage of BODIPY-labeled hC5a by ScpA, D783A, E864A and D889A ScpA mutants respectively. Data are plotted with curves from global fitting of curves (black lines) to the Van Slyke-Cullen model modified to account for product inhibition. Values for *k*_cat_ and *K*_m_ are reported as the mean and standard deviation of the mean from 4 experiments. Data shown in the plot are from 4 experiments. A key to substrate concentrations is provided in panel e. Panel (f) Van Slyke-Cullen mechanism modified for product inhibition used in fitting of progress curves. Van Slyke-Cullen kinetic parameters were used to obtain steady state enzyme kinetic parameters (*K*_m_ = *k*2/*k*1 and *k*_cat_ = *k*2).Panel (g) shows a reaction scheme where binding of full-length hC5a (‘S’) occurs with a conformational change in ScpA (‘E’ to ‘F’). Following acylation, release of the C-terminal ‘tail’ (‘P_C_’) and deacylation steps, the core portion of hC5a (‘PN’) is released from conformational state ‘F’. These rates are fast as compared to release of the ‘PN’ product from the ‘E’ ScpA state measured in SPR studies.

Substrate cleavage by the D783A enzyme was significantly slower than the wild-type enzyme (Fig. 4b). The *k*_cat_ for D783A is approximately 45% lower than for ScpA (*k*_cat_ = 0.554 s^−1^) while the *K*_m_ increased by approximately 3-fold (*K*_m_ = 560 nM). The increase in *K*_m_ for D783A is consistent with the lower binding affinity observed for substrate binding in the SPR studies (Section 3.3). However, while the impact of the D783A mutation on *K*_m_ might be expected, the decrease in *k*_cat_ observed for this mutant is completely unexpected given mutation occurs at a site more than 48 Å from the catalytic site. The significant impacts on substrate binding and C5a-ase activity prompted an investigation into the structure of D783A and the crystal structure of the active form of the D783A was solved to 1.9 Å resolution (See Data in Brief). The mutant structure is nearly identical to the wild-type ScpA with low RMSDs between Cα atoms (0.457 Å) and atoms in the catalytic residues (0.136 Å). Thus, the decrease in substrate binding affinity and activity is not the result of a change in the fold of the enzyme.

The enzyme kinetic parameters determined for E864A and D889A are similar to that for the wild-type form of ScpA (Fig. 4c and d respectively). As with the D783A mutant, the *K*_m_ values reflect the general trend observed for substrate binding as determined by SPR (Section 3.3). Of the examined ScpA forms, the D783A enzyme exhibited the lowest *k*_cat_ which is inconsistent with the apparent enhanced cleavage efficiency observed in the end-point activity assay (Section 3.2). Based on the observed *K*_m_s all enzymes would be turning over substrate at near maximum velocity in the presence of 18 µM substrate and thus D783A should be the least efficient at cleaving the substrate. However, this interpretation does not consider the product inhibition observed in the enzyme kinetic analysis. SPR studies with rhC5a_core_ (Section 3.4) showed that the D783A mutant bound the product with much lower affinity than the other ScpA forms (11.6–24.6-fold higher *K*_D_). Thus, the apparent higher efficiency of substrate cleavage by D783A reflects decreased product inhibition in the end-point activity assay.

The enzyme kinetic analysis of the ScpA mutants supports the mechanism presented previously [10], and shown in Fig. 4g. All forms of the enzyme have a much higher *k*_cat_ than the rate of release of the rhC5a_core_ product (‘P_N_’) measured by SPR (Section 3.4). The *k*_d_ for rhC5a_core_ binding to ScpA, D783A, E864A and the D889A mutants (Section 3.4) is 134-, 26-, 290- and 231-fold slower than the *k*_cat_ although apparently not rate limiting. Previously, this discrepancy was explained by suggesting that the rhC5a_core_ interaction studied by SPR was not involved the catalytic mechanism [10]. Rather, the SPR analysis of the rhC5a_core_ interaction with ScpA and its mutants characterize the interaction associated with product inhibition and that efficient release requires a conformational state (‘F’) adopted only in the presence of the tail residues.

### 3.6 Double mutant cycle analysis indicates energetic coupling between the ScpA Fn2 domain and the core portion of C5a

The examination of single point mutations on binding provides information on the energetic contribution of the residue to the stability of the complex (ΔG°_bind_). Double mutant cycle (DMC) analysis of the impact of two mutations simultaneously can reveal more complex details of a process, such as cooperativity or energetic coupling between residues [20], [21]. For inter-molecular interactions, DMC analysis involved pair-wise characterization of single mutations in both binding partners. Additivity or non-additivity of ΔG°_bind_ for single mutations as compared to both mutations simultaneously reveal the extent of communication between the residues [22].

**Fig. 5:**
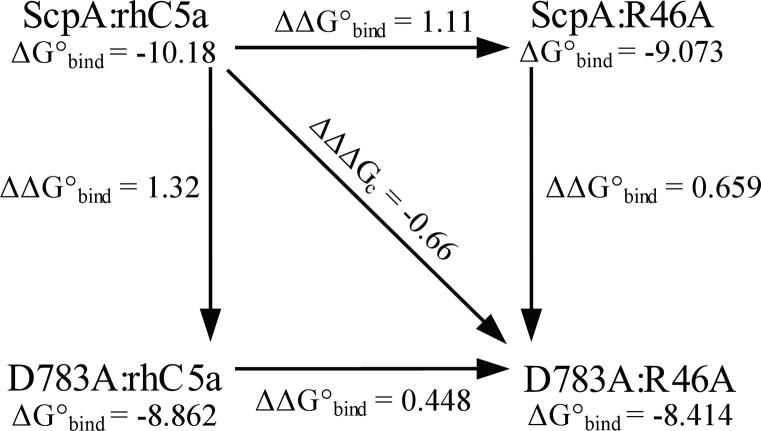
Double mutant cycle analysis of ScpA and rhC5a mutants. A representative example of the analysis cycle accompanying calculation of coupling energies is shown for ScpA residue D783 and rhC5a residue R46.

**Supplementary figure 5:**
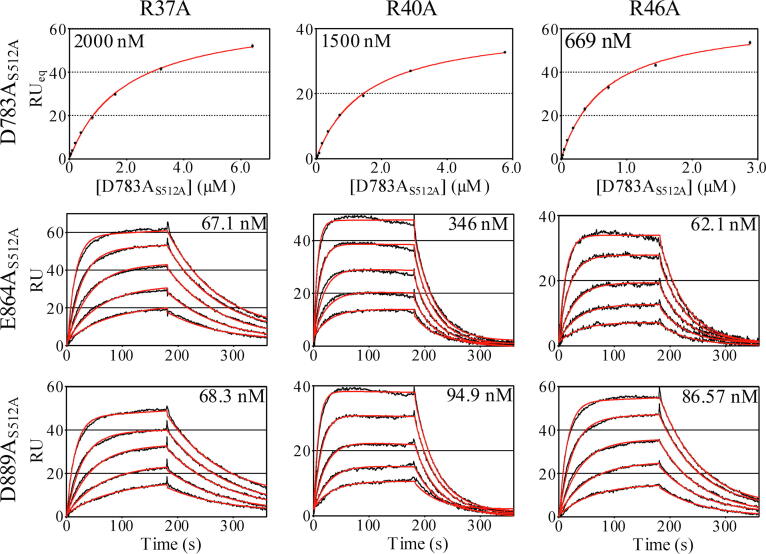


DMC analysis was applied to the pairwise coupling of residues D783, E864 and D889 in ScpA with residues R37, R40 and R46 of the full-length rhC5a (for example see Fig. 5). The sensorgrams for these analyses are shown in Fig. S5. Coupling energies between residue pairs (ΔΔΔG_C_ on Table 4) were obtained using Eq. (2) with the binding energies (ΔG°_bind_) reported on Table 2. In Table 4, coupling energies considered to be comparable in magnitude are highlighted in red and blue (negative and positive values respectively). Coupling energies near zero are not highlighted. For substrate binding (top half of Table 4) non-zero ΔΔΔG_C_ are observed for 8 of the 9 pairs examined ranging from −0.66 to +0.29 kcal/mol. The majority of the coupling energies (7 of 9) are negative in sign except for the D783:R37 and E864:R40 pairs which have near zero and positive ΔΔΔG_C_s, respectively.

**Table 4:** Coupling energies (ΔΔΔG_c_)^a,b^ for ScpA and rhC5a mutants.

Interestingly, while the SPR analysis (Section 3.3) showed that only the D783A mutation impacted directly on substrate binding, the DMC analysis indicates that all three ScpA residues are energetically coupled to residues in the C5a core in the enzyme:substrate complex. This lends additional support for the involvement of the Fn2 domain in substrate interactions and suggests that residues across the Fn2 domain participate in the process of substrate binding. This could involve roles such as the stabilization or communication of structural transitions in the enzyme and/or substrate.

### 3.7 DMC analysis of the ScpA:product (C5a_core_) interaction supports a distinct mode for product binding

DMC analysis was also used to estimate coupling energies between mutated residues in ScpA and the C5a core during product binding. The sensorgrams for these analyses are shown in Fig. S6 and the binding parameters and ΔG°_bind_ are reported on Table 3. The ΔΔΔG_C_ values obtained in the DMC analysis of the binding to the rhC5a_core_ mutants are reported in the lower half of Table 4.

The ΔΔΔG_C_ values for product binding range from −0.37 to 0.57 kcal/mol. Non-zero ΔΔΔG_C_ values are observed for 6 of the 9 pairs examined indicating, as with the substrate, Fn2 residues are energetically coupled to residues in the core of the product. In contrast to binding mutants of the substrate, the coupling energies for product binding are predominantly (5 of 9) positive in value. This strikingly different pattern suggests that the process of binding and recognition of the substrate is different from that for the product. Competitive binding studies have established that substrate and product compete for an overlapping site on ScpA [10]. However, data in this study supports previous assertions that the modes of binding for the substrate and the product are distinct [10].

## 4 Discussion

An important new strategy for developing therapeutic interventions for immune dysfunction disorders is to target the complement cascade (recently reviewed in [23]). One of the emerging candidate technologies is the exploitation of bacterial proteases. To support this approach, a better understanding of the contribution of the distinct domains in enzymes such as ScpA to specificity and activity is required.

The current study directly addresses whether the Fn2 domain in ScpA is involved in the substrate interactions. In preliminary screening of deletion mutants with sequential deletions of the three C-terminal fibronectin type III domains (Fn1, Fn2 and Fn3 domains) (Section 3.1), the presence of the Fn2 domain was required for activity. The only construct retaining C5a-ase activity was the mutant with an Fn2 domain (ScpAΔFn3). Interestingly, substrate hydrolysis could be recovered by the inactive mutants (ScpAΔFn123 and ScpAΔFn23) when the Fn2 domain was provided separately as independently folded entities (Fn123 or Fn23). Substrate cleavage when ScpAΔFn123 was complemented with Fn123 suggesting that C5a is delivered to the Cat domain via the formation of a ternary complex between the 2 ScpA entities and the substrate. However, an additional off-target cleavage sites was revealed between V57 and A58 indicating that presentation of the substrate to the active site was non-native-like. The activity observed with Fn123 complementation was approximately 0.1% of the wild-type enzyme (data not shown), suggesting a weak interaction between Fn123 and ScpAΔ123. In the structure of ScpA, a loop in the catalytic domain (referred to as the hammerhead loop) is inserted between the Fn1 and Fn2 domains. It is likely that this structural organization cannot be achieved when the two ScpA constructs are combined as separately folded entities impacting on the delivery of the substrate. Weak activity of ScpAΔFn23 was restored by addition of either Fn123 or Fn23. Analysis of the products in both reactions showed that cleavage occurs between V57 and A58. The mechanism by which this is achieved requires further investigation. Notwithstanding, the observations from these complementation studies indicates the Fn2 domain is involved in substrate interactions.

In SPR studies, the impact of 3 point mutations at D783, E864 and D889 in the Fn2 domain were examined (Section 3.3). The D783A mutation decreased ΔΔG°_bind_ by 1.32 kcal/mol relative to the wild-type interaction with an approximately 9-fold increase in *K*_D_. Mutation of residues E864 and D889 did not appear to significantly impact the interaction of ScpA with C5a (Table 1 and Fig. 4). In the enzyme kinetic analysis, the D783A mutant exhibited a nearly 3-fold increase in the *K*_m_ (189 to 560 nM) consistent with the decrease in substrate binding affinity observed in the SPR studies. In addition, a 1.8-fold lower *k*_cat_ was observed relative to ScpA (0.554 vs 1.006 s^−1^). The crystal structure of the D783A mutant showed that the mutation did not disrupt the overall fold of the enzyme or the geometry of the catalytic residues (Data in Brief) indicating that the changes in binding and enzyme kinetic properties of the mutant are related to alterations in interactions involving residue D783. Thus, these studies provide direct evidence that the Fn2 domain participates in determining the specificity and activity of ScpA and is not simply a structural component of the enzyme. While it is relatively easy to rationalize the impact of the D783A mutation on *K*_m_ or *K*_D_ as resulting from the elimination of a significant interaction with the substrate, it is less straightforward to explain the effect of this mutation on the ability to turn over the substrate. It could be argued that the rate of turnover is limited by a slower rate of product release. However, this view is not supported by the SPR data on the rhC5a_core_ interactions which shows that product release is expected to be faster with the D783A mutant as compared to the wild-type form of ScpA (Section 3.4). As mentioned previously, these measurements do not apply to the catalytic cycle since they probe an inhibitory interaction with the product (Section 3.4). Additional studies that consider long-range interdomain communication will be required to examine how a region distal to the catalytic site is involved in the chemical transformation of the substrate. Unravelling the details of how the Fn2 domain participates in both substrate binding affinity and enzymatic activity could allow the uncoupling of these two properties and reveal a general strategy for optimizing ScpA for other targets. For example, ScpA has been demonstrated to cleave both C3a and C5a [7]. Since the physiological concentration of C3a has been reported to be higher than C5a (20–40 nM vs 1–10 nM [24]), identifying an ScpA mutant selective for C3a may involve increasing the *K*_m_ for C5a above a threshold, while retaining or enhancing its ability to efficiently cleave C3a.

A double mutant cycle analysis was performed to probe the coupling between the 3 ScpA residues and 3 C5a residues (Fig. 1A). Non-zero coupling energies (ΔΔΔG_C_) were observed between all ScpA and C5a residues tested except for the D783:R37 pair, indicating that the majority of the examined residue pairs are functionally linked in the binding process. Thus, despite substrate ΔΔG°_bind_ values near 0 for the E864A and D889A mutants, the DMC analysis supports the contribution of these residues to substrate binding. This suggests that the region of the exosite involved in substrate binding is extensive since the ScpA residues examined are located at the periphery of the Fn2 domain separated by approximately 20–35 Å. Thus, the limited DMC analysis supports the existence of a complex communication network between the enzyme and the substrate with residues distributed across the Fn2 domain energetically coupled to residues located between Helix III and IV in the core of C5a.

Additional information from DMC analysis can be obtained from the sign of ΔΔΔG_C_ which has been suggested to indicate the extent of energetic optimization between residue pairs. Negative ΔΔΔG_C_ values were proposed to be associated with an un-optimized binding pair while optimized interactions would exhibit positive values [22]. The DMC analysis of ScpA:substrate interaction identified a single optimized residue pair, D864:R40. 7 of the 8 remaining pairs exhibited negative ΔΔΔG_C_s indicating that these residue pairs are not optimized. It is possible that this property is related to the ability of ScpA to inactivate both C5a and C3a which have a common 3-dimensional fold but differ in sequence (34% identity).

The inhibitory interaction between ScpA and the core portion of C5a (rhC5a_core_) was also examined with a DMC analysis (Section 3.7). These studies characterize binding between ScpA and the larger *N*-terminal product of proteolysis (P_N_). As with substrate binding, non-zero ΔΔΔG_C_s were observed for all 3 ScpA residues. However, the signs for the majority of ΔΔΔG_C_ values are positive (5 of 9) and coupling energies near zero are observed for pairings with the R40A mutant. Thus, the DMC analysis shows that the residue interactions are better optimized for product binding. This provides additional support for distinct ScpA modes for binding the substrate versus product (Fig. 4g) reported by Teçza et al. [10].

The magnitude of the ScpA:substrate coupling energies (−0.66 to +0.29 kcal/mol) are on the order of those reported by others for protein–protein interactions [21], [22]. However, little further information can be inferred as the relationship between the magnitude of the coupling energy and distance between coupled residues is unresolved in the literature [22], [25]. Assignment of interacting pairs in the ScpA:C5a complex will benefit from structural studies. In addition, interdomain communication networks in supertertiary structures have been associated with negative values for coupling energies in substrate binding [21], such as seen in the DMC for the enzyme:substrate complex here. Thus, given the potential complexity of the interaction, additional studies are required to investigate the existence of similar networks or allosteric systems in ScpA. Expansion of the DMC analysis could reveal coupling with residues in other domains and allow the exploration of pathways important for gating specificity.

## 5 Concluding remarks

Cell envelope proteases have been identified in Gram positive commensals of the human gut [26] as well as in a variety of streptococcal species (reviewed in [13]) that potentially encounter mediators of the host immune response. In a homology search of the protein sequences available in the current Ensembl bacteria database, approximately 1500 non-redundant sequences were identified that have the basic CEP architecture (Kagawa and Cooney, unpublished). We have proposed ScpA as a prototype for engineering therapeutic proteases that inactivate immunomodulatory proteins based on its high specificity for human complement C5a and C3a, along with the availability of methodologies for studying structure and now function of this enzyme [10]. Essential to exploitation of proteolytic enzymes as catalytic biologics is understanding how they function at a molecular level. Toward this end, we have shown that binding of the substrate includes Arginine residues in tail and core of C5a implicating ScpA residues both in and distal to the active site in substrate interactions [10]. The current studies indicate that residues in the ScpA Fn2 domain, in particular residue D783, are involved in enzyme:substrate interactions, thus providing experimental support for the existence of an exosite on the Fn2 domain. Interestingly, residue D783, which is nearly 50 Å from the catalytic serine was also found to impact on the ability of the enzyme to turn over the substrate. While the results presented here indicate D783 is directly involved in substrate interactions they do not exclude alternative interpretations that support a more complex binding and recognition process. For example, the mutations in Fn2 could disrupt interdomain contacts that stabilize a conformational state required for binding of the substrate and or efficient progression through the catalytic cycle. Taken together with the suggested role of the PA domain in active site interactions [13], our results indicate the potential for coupling of substrate selection and hydrolysis by long range communication mechanism involving different conformational states in ScpA. The involvement of alternative conformational states in the specificity and activity of ScpA would introduce the intriguing possibility of tuning the enzyme by manipulating residues or clusters of residues involved in the transition between these states.

## CRediT authorship contribution statement

**Monica Jain:** Investigation, Methodology, Formal analysis, Writing – original draft. **Malgorzata Teçza:** Investigation, Methodology, Formal analysis, Writing – original draft. **Todd F. Kagawa:** Investigation, Methodology, Conceptualization, Formal analysis, Writing – original draft, Writing – review & editing, Supervision. **Jakki C. Cooney:** Investigation, Methodology, Conceptualization, Formal analysis, Writing – original draft, Writing – review & editing, Funding acquisition, Supervision.

## Declaration of Competing Interest

The authors declare the following financial interests/personal relationships which may be considered as potential competing interests: JCC and TFK are inventors on a patent for the development of ScpA as a therapeutic for sepsis, licenced to a company through the University of Limerick.

## Figures and Tables

**Supplementary figure 6:**