# Supplementary material for: Exosite binding modulates the specificity of the immunomodulatory enzyme ScpA, a C5a inactivating bacterial protease
Source: Comput Struct Biotechnol J. 2022 Aug 27;20:4860–9. doi: 10.1016/j.csbj.2022.08.018 (PMC9464890; doi:10.1016/j.csbj.2022.08.018)
Supplement: Supplementary data 1 [file mmc1.docx]

**Supplemental information**

**SI1. Preparation of Recombinant Proteins**

Domain drop-out mutations (DDOs) in ScpA were generated using overlapping PCR fragments flanking the region to be deleted. Resultant amplicons were cloned into pGEX-6P-3 for expression of proteins in *E. coli*. Purification of recombinant ScpA drop-out mutations followed the standard purification protocol established for the wild-type ScpA and ScpA_S512A_. Purity and integrity of the isolated proteins was evaluated using SDS-PAGE and CD spectropolarimetry.

Stability of purified proteins varied. Forms of the protein with a wild-type active site (ScpAΔFn3 and ScpAΔFn23) were purified with at least 60% of the dominant entity being the required form. The versions of ScpA comprising only Fn23 or Fn123 domains were produced as homogenous preparations (Fig S1). All proteins with the active site mutation (S512A) which require treatment with SpeB during production, were unstable.

Production of recombinant human C5a peptides, point mutations and cores used in this study has been previously described [1].

**
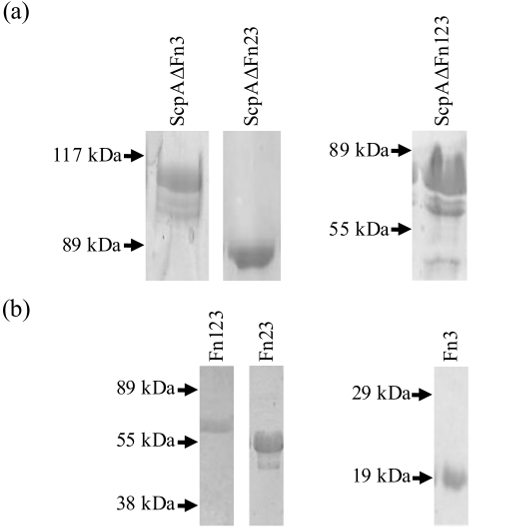
**

**Fig. S1. Purification of Domain Drop-out (DDO) mutations of ScpA.**

Panel (a) shows an SDS-PAGE analysis of the purified variants of ScpA which contain a catalytic domain. Panel (b) shows an SDS-PAGE analysis of the purified variants of ScpA which contain only Fn domains.

**SI2. Assessment of protein stability for ScpA point mutations by CD spectropolarimetry and differential scanning fluorimetery.**

The stability of ScpA and its variants were assessed with circular dichroism (CD) and differential scanning fluorimetry (DSF). For CD measurements, the purified proteins were dialyzed into 2 mM potassium phosphate pH 7.4. Protein concentrations were calculated from the absorbance at 205 nm and sequence specific molar absorptivity [2]. The concentrations ranged between 13-19 µg/mL. CD spectra were obtained on a Chirascan V100 spectropolarimeter (Applied Photophysics, UK) with a scan rate of 4 sec per nm. Detector HV remained below 600 V. Raw CD measurements (mdeg) were converted to Δε units (M^-1^ cm^-1^) for deconvolution with CDSSTR program of the CDPro software package [3]. CD measurements and fitted curves are shown in Fig S2a. No difference in secondary structure content of the ScpA proteins was detectable using CD, however the α-helical content was consistently and equally under-determined by CD when compared to the solved structures.

Thermal stability of the proteins was measured with DSF (also known as the Thermofluor assay). Purified proteins (~1 mg/mL in PBS) were combined with a Sypro Orange (Merck, UK) solution diluted 1000-fold into PBS. Fluorescence was measured on a LightCycler 480ii Real-Time PCR system (Roche, USA) with excitation at 498 nm and emission at 610 nm, as the samples were heated from 25 °C to 95 °C with a ramp rate of 0.03 °C/sec and 20 data acquisitions per °C. Melting curves for each protein were measured in triplicate. The melting temperature (T_m_) was estimated with a first derivative analysis to identify the point of maximum slope in the transition curve. The data and were scaled by the highest fluorescence value and plotted with T_m_s in Fig S2b. Table S1 report the average and standard deviation from the three determinations of T_m_.

**
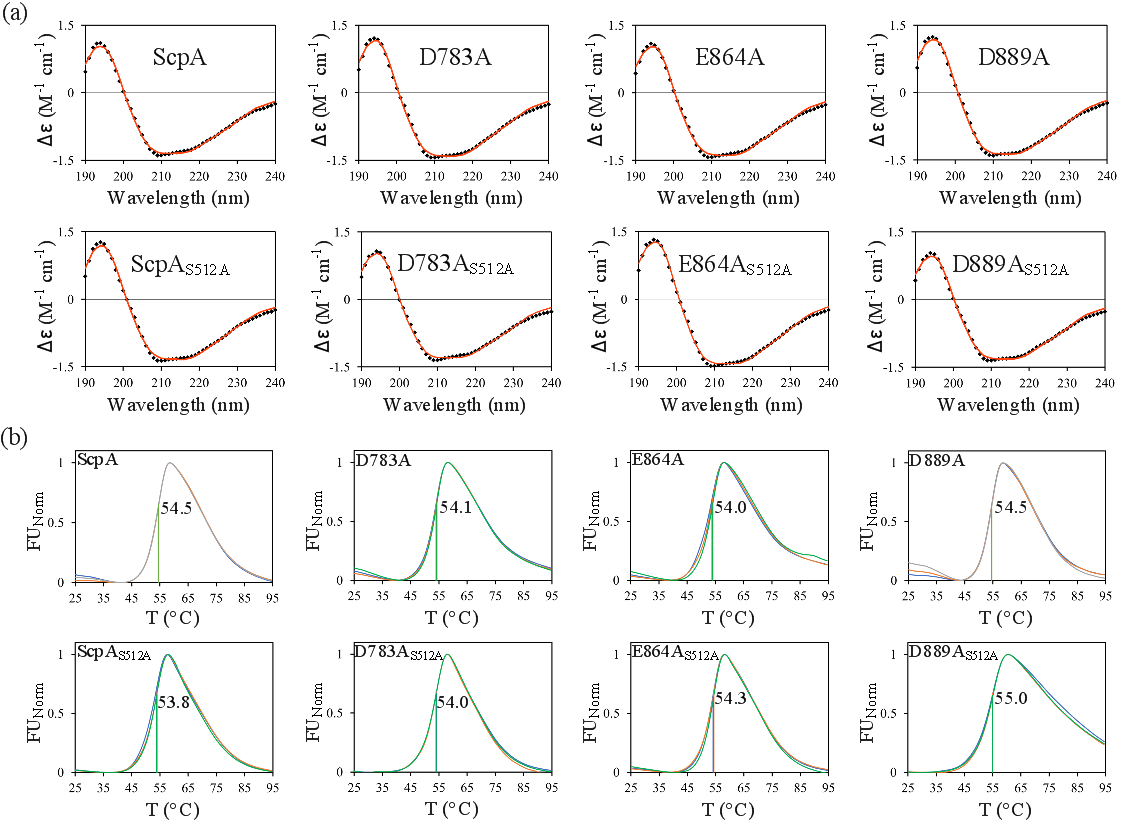
**

**Fig. S2 Assessment of protein stability for ScpA point mutations by CD spectropolarimetry and differential scanning fluorimetery.**

CD data (a) shown as black triangles and fitted curves as red lines. The fitted curves were obtained by deconvoluting the observed data with CDSSTR. Melting curves (b) of ScpA proteins shows data from 3 experiments. Melting temperature (T_m_) from a first-derivative analysis are shown with vertical lines. The average Tm are reported on the plots.

Table S1. Melting temperatures and secondary structural content of ScpA proteins[4]

| **Protein** | **Tm (°C)** | **%helix** | **%sheet** | **%helix^*^** | **%sheet^*^** |  |
| --- | --- | --- | --- | --- | --- | --- |
| ScpA^1^ | 54.5 ± 0.1 | 5 | 38 | 18.5 | 31.1 |  |
| D783A^2^ | 54.1 ± 0.1 | 7 | 38 | 18.5 | 30.7 |  |
| E864A | 54.0 ± 0.1 | 7 | 38 |  |  |  |
| D889A | 54.5 ± 0.1 | 7 | 38 |  |  |  |
| ScpA_S512A_^3^ | 53.8 ± 0.1 | 6 | 37 | 18.7 | 31.1 |  |
| D783A_S512A_ | 54.0 ± 0.0 | 6 | 37 |  |  |  |
| E864A_S512A_ | 54.3 ± 0.1 | 7 | 37 |  |  |  |
| D889A_S512A_ | 55.0 ± 0.0 | 7 | 37 |  |  |  |
| ^1^ ScpA PDB ID 3 EIF. ^2^ D783A PDB ID 7YZX. ^3^ ScpA_S512A_ PDB ID 7BJ3. | | | | |  |  |
| ^*^ Calculated from structure with 2Struct server [4] using the DSSP method | | | | |  |  |


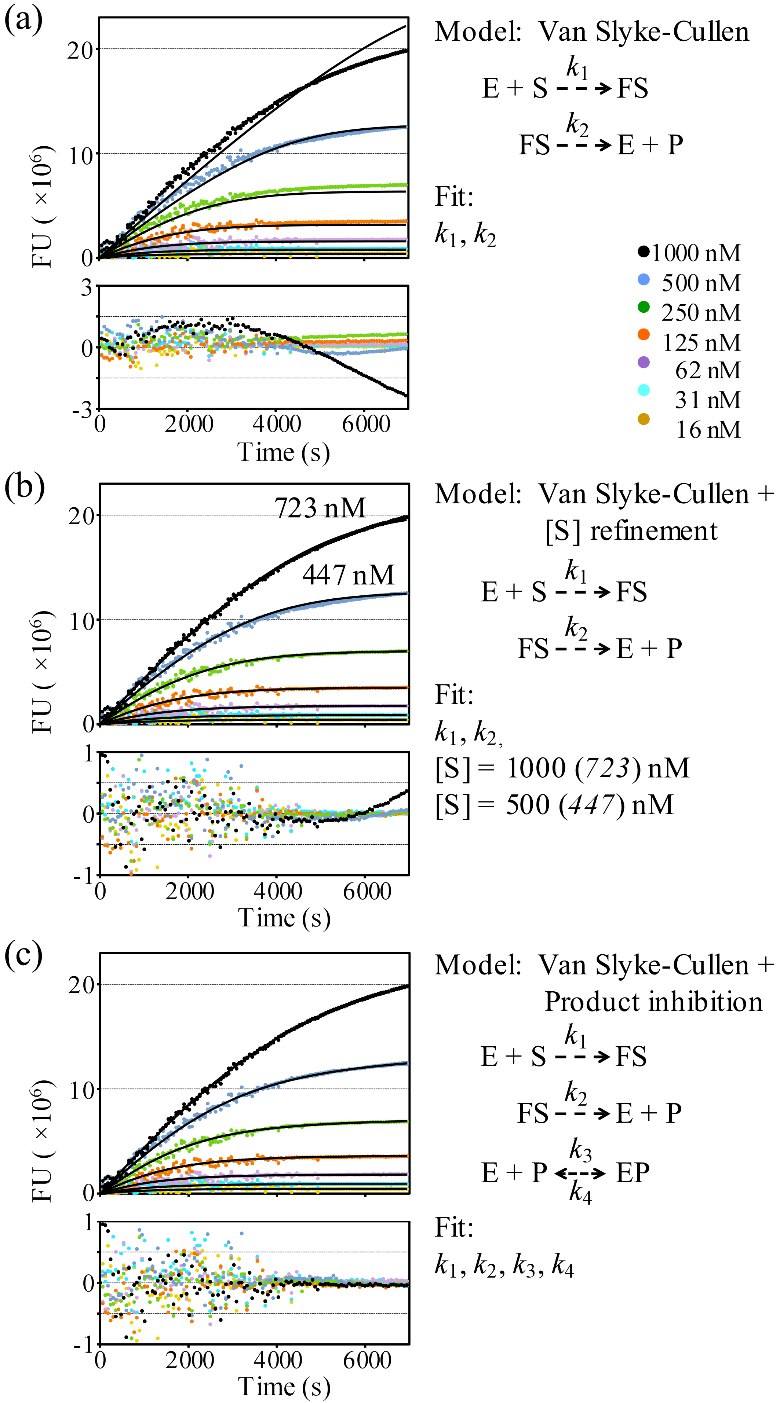
**Fig. S3 Progress curve fitting with product inhibition.**

The 3 strategies tested for fitting enzyme kinetic progress curves. Progress curves are shown with coloured circles, and the fitted curves as black lines. For all 3 panels, global fits of the data are shown in the top plot and the residual plots below. The colouring scheme for the data is shown in Fig. S5a. Panel (a) shows results from data fitted with the Van Slyke-Cullen mechanism. The model for this strategy shows the formation of the Michaelis complex (‘FS’) from enzyme (‘E’) and substrate (‘S’) followed by product formation (‘P’) and regeneration of the enzyme. Large systematic residuals are observed at later time points for fitting of the 1000 nM data with this fitting protocol. Panel (b) shows reduced residuals when including refinement of substrate concentrations ([S]) for the 1000 and 500 nM data. Refined values for [S] are shown in parentheses and reported on the plot. The model used in the fitting shown in Panel (c) includes parameters to account for product inhibition (*k*3 and *k*4). The improved fit to the data does not require refinement of [S].

Table S2. Top concentration of ligate used in SPR experiments^*^.

| Ligand  Ligate | rhC5a | R37A | R40A | R46A | rhC5a_core_ | R37A_core_ | R40A_core_ | R46A_core_ |
| --- | --- | --- | --- | --- | --- | --- | --- | --- |
| ScpA_S512A_ | 180 | 720 | 720 | 720 | 720 | 1600 | 3200 | 3200 |
| D783A_S512A_ | 1600 | 6400 | 5760 | 2880 | 6400 | 11500 | 11500 | 11500 |
| E864A_S512A_ | 180 | 400 | 1440 | 180 | 400 | 1600 | 3200 | 1600 |
| D889A_S512A_ | 180 | 400 | 360 | 400 | 400 | 1600 | 3200 | 3200 |

^*^Reported in nM

**
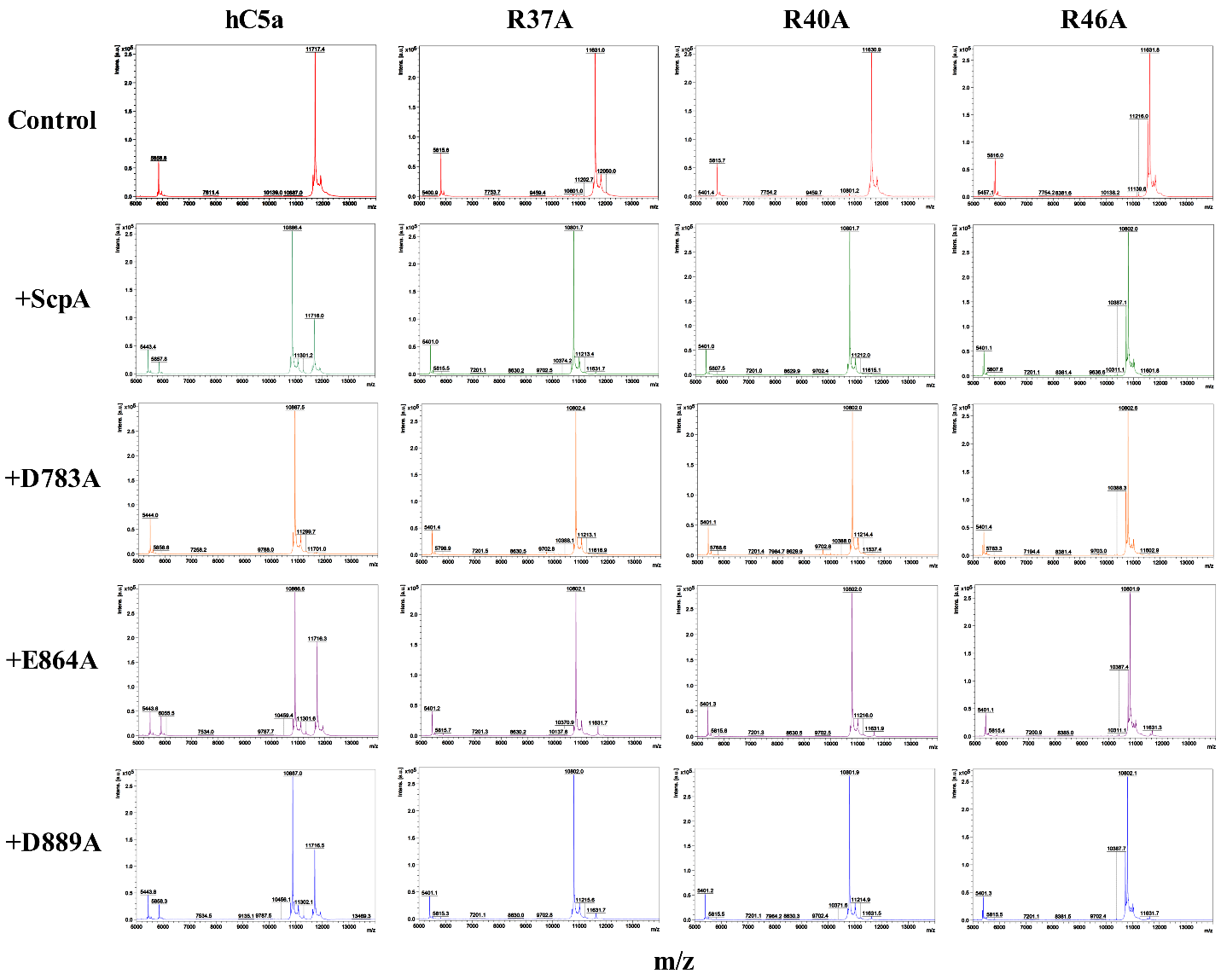
**

**Fig. S4 Mass spectrometry of rhC5a substrates cleaved by ScpA enzymes.** Mass spectrometry was used to characterize cleavage products produced by ScpA and ScpA mutants following the procedure reported previously [1]. Each column is a set of mass spectra for the wild-type or a mutant rhC5a as indicated. The top row is a set of controls where the different C5a proteins were untreated. The subsequent rows are C5a treated with the indicated form of ScpA. The values are tabulated in Supplemental Table S2.

Table S3: Mass spectrometry data on cleaved hC5a and hC5a mutants

| **Sample** | **Calculated mass (Da)^1^** | **Observed mass (Da)** | **Difference (Da)^2^** |
| --- | --- | --- | --- |
| hC5a | 11715.25 | 11717.40 | NA |
| R37A | 11630.14 | 11631.00 | NA |
| R40A | 11630.14 | 11630.90 | NA |
| R46A | 11630.14 | 11631.80 | NA |
| hC5a + ScpA | 10886.27 | 10886.40 | 831.00 |
| R37A + ScpA | 10801.16 | 10801.70 | 829.30 |
| R40A + ScpA | 10801.16 | 10801.70 | 829.20 |
| R46A + ScpA | 10801.16 | 10802.00 | 829.80 |
| hC5a + D783A | 10886.27 | 10887.50 | 829.90 |
| R37A + D783A | 10801.16 | 10802.40 | 828.60 |
| R40A + D783A | 10801.16 | 10802.00 | 828.90 |
| R46A + D783A | 10801.16 | 10802.60 | 829.20 |
| hC5a + E864A | 10886.27 | 10886.60 | 830.80 |
| R37A + E864A | 10801.16 | 10802.10 | 828.90 |
| R40A + E864A | 10801.16 | 10802.00 | 828.90 |
| R46A + E864A | 10801.16 | 10801.90 | 829.90 |
| hC5a + D889A | 10886.27 | 10887.00 | 830.40 |
| R37A + D889A | 10801.16 | 10802.00 | 829.00 |
| R40A + D889A | 10801.16 | 10801.80 | 829.10 |
| R46A + D889A | 10801.16 | 10802.10 | 829.70 |

^1^ Masses calculated with Protparam [5]. 70 Da added to account for βME bound a C27 of hC5a and mutants

^2^ The difference is the loss in mass upon treatment with enzymes. Cleavage of the 7 C-terminal tail residues of C5a results in a loss of 829 Da.


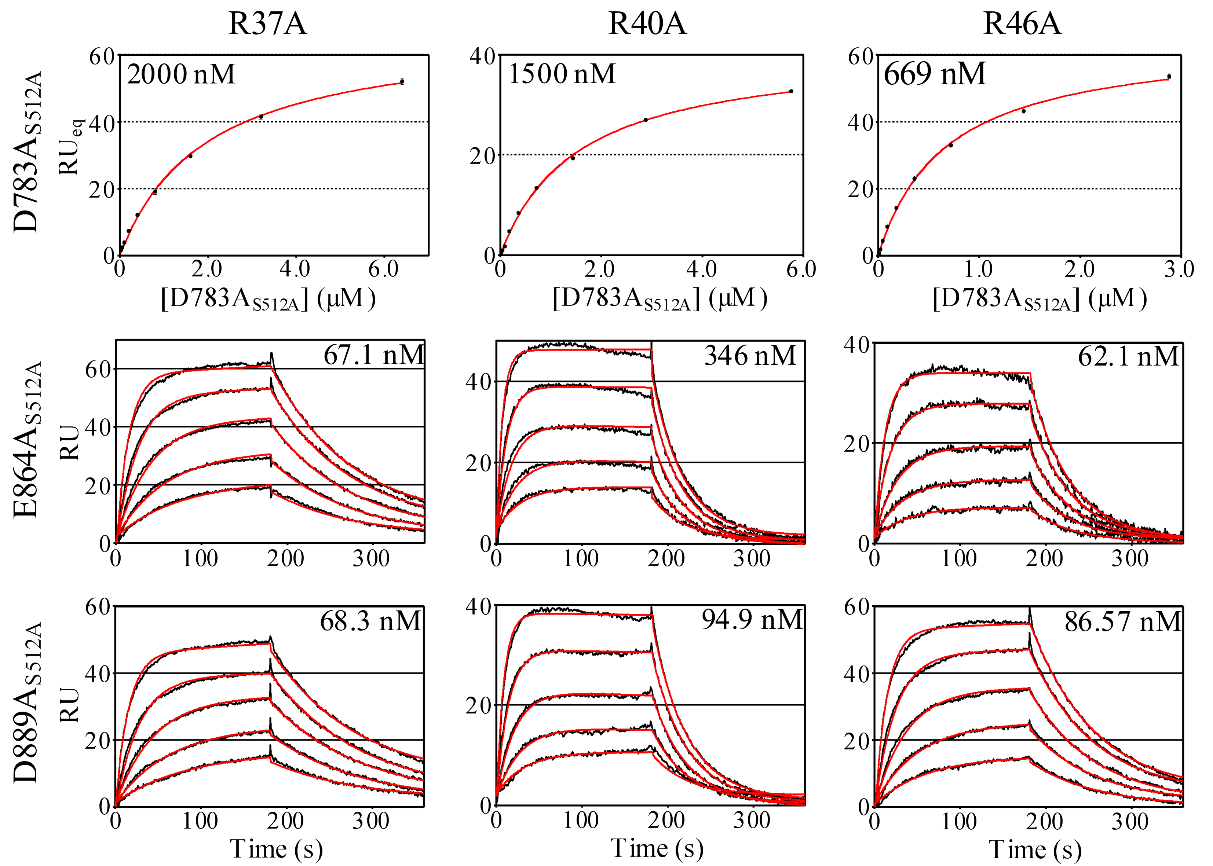


**Fig S5 SPR sensorgrams for double mutant analysis of ScpA mutants binding with rhC5a mutants.**

Representative SPR sensorgrams of ScpA_S512A_ and ScpA_S512A_ mutants binding to immobilized full-length rhC5a and mutant derivatives. Observed data for the E864A_S512A_ and D889A_S512A_ mutants (black lines) are shown with curves obtained from global fitting of data with a 1:1 Langmuir model for binding (red lines). For the D783A_S512A_ mutant, the equilibrium response units (RU_eq_) were fit with the steady state affinity model in the BiaEvaluation software to obtain the equilibrium dissociation constant for binding. The mean *K*_D_ value obtained from 3 experiments is reported in the upper right-hand corner of the respective panel.


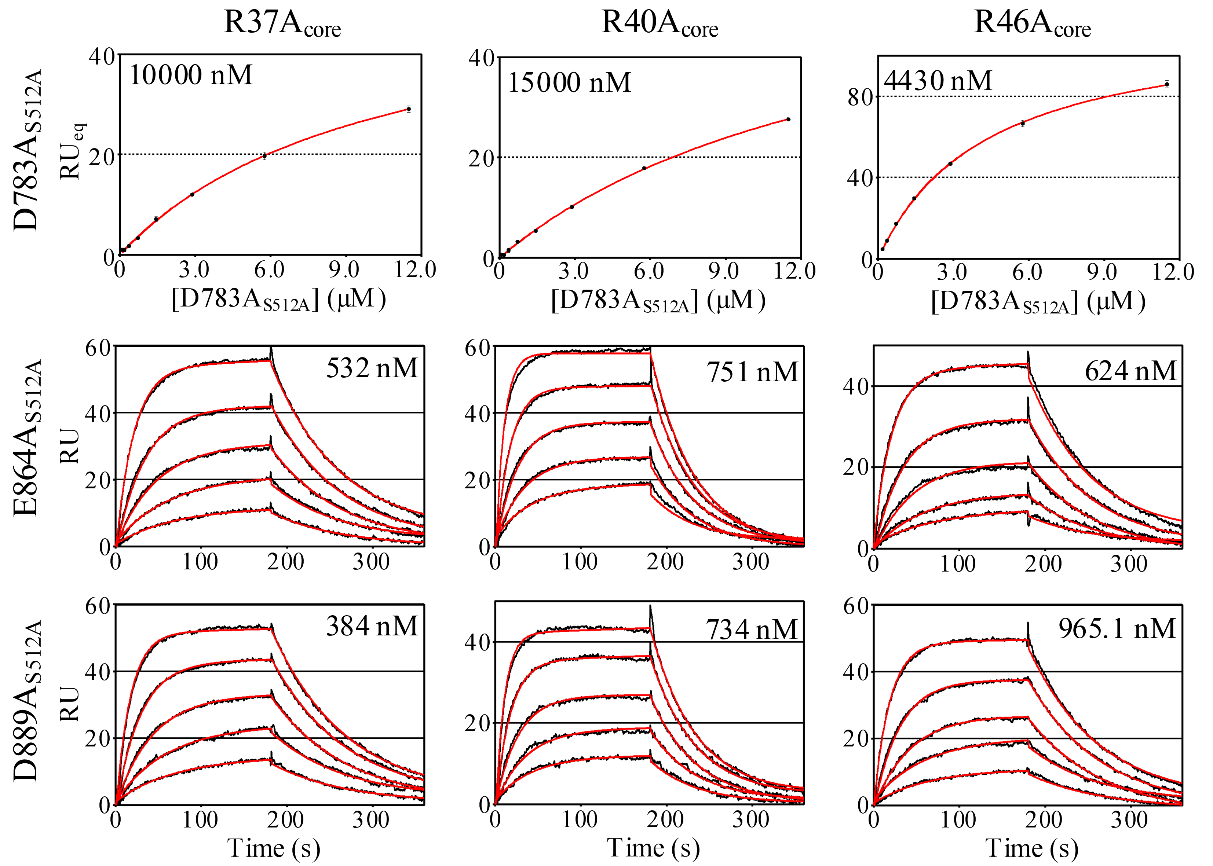


**Fig S6 SPR sensorgrams for double mutant analysis of ScpA mutants binding with rhC5a_core_ mutants.**

Representative SPR sensorgrams of ScpA_S512A_ and ScpA_S512A_ mutants binding to immobilized rhC5a_core_ and mutant derivatives. Observed data for the E864A_S512A_ and D889A_S512A_ mutants (black lines) are shown with curves obtained from global fitting of data with a 1:1 Langmuir model for binding (red lines). For the D783A_S512A_ mutant, the equilibrium response units (RU_eq_) were fit with the steady state affinity model in the BiaEvaluation software to obtain the equilibrium dissociation constant for binding. The mean *K*_D_ value obtained from 3 experiments is reported in the upper right-hand corner of the respective panel.

**References**

1. Tecza, M., et al., *Enzyme kinetic and binding studies identify determinants of specificity for the immunomodulatory enzyme ScpA, a C5a inactivating bacterial protease.* Comput Struct Biotechnol J, 2021. **19**: p. 2356-2365.

2. Anthis, N.J. and G.M. Clore, *Sequence-specific determination of protein and peptide concentrations by absorbance at 205 nm.* Protein Sci, 2013. **22**(6): p. 851-8.

3. Sreerama, N. and R.W. Woody, *Estimation of protein secondary structure from circular dichroism spectra: comparison of CONTIN, SELCON, and CDSSTR methods with an expanded reference set.* Anal Biochem, 2000. **287**(2): p. 252-60.

4. Klose, D.P., B.A. Wallace, and R.W. Janes, *2Struc: the secondary structure server.* Bioinformatics, 2010. **26**(20): p. 2624-5.

5. Wilkins, M.R., et al., *Protein identification and analysis tools in the ExPASy server.* Methods Mol Biol, 1999. **112**: p. 531-52.
